# Synthetic Approaches to Novel DPP-IV Inhibitors—A Literature Review

**DOI:** 10.3390/molecules30051043

**Published:** 2025-02-25

**Authors:** Valentin Petrov, Teodora Aleksandrova, Aleksandar Pashev

**Affiliations:** Department of Chemistry and Biochemistry, Faculty of Pharmacy, Medical University—Pleven, 1 St. Kliment Ohridski Str., 5800 Pleven, Bulgaria; vali.petrov@abv.bg (V.P.); teodora.aleksandrova@mu-pleven.bg (T.A.)

**Keywords:** DPP-IV inhibitors, type 2 diabetes, heterocyclic synthesis, drug design, medicinal chemistry

## Abstract

Dipeptidyl peptidase IV (DPP-IV) is a serine protease whose inhibition has been an object of considerable interest in the context of developing novel treatments for type 2 diabetes mellitus. The development of novel DPP-IV inhibitors from natural or synthetic origin has seen a growing scientific interest in recent years, especially during the SARS-CoV-2 pandemic, when DPP-IV inhibitors were found to be of beneficial therapeutic value for COVID-19 patients. The present manuscript aims to summarize the most recent information on the synthesis of different DPP-IV inhibitors, emphasizing the various heterocyclic scaffolds that can be found in them. Special attention is devoted to DPP-IV inhibitors that are currently in clinical trials. Different synthetic approaches for the construction of DPP-IV inhibitors are discussed, as well as the most recent developments in the field.

## 1. Introduction

Dipeptidyl peptidase IV (DPP-IV) can be regarded as a promising therapeutic target in the treatment of type 2 diabetes mellitus [1,2,3,4,5,6]. DPP-IV is involved in the metabolism of incretin hormones (GLP-1 and GIP) in the body, which regulate insulin secretion [7]. Currently, there are twelve drugs that act as DPP-IV inhibitors and have been approved by regulatory agencies. DPP-IV inhibitors attracted even greater scientific interest during the SARS-CoV-2 pandemic, when their ability to mitigate the severity of COVID-19 disease was discovered [8,9,10,11,12]. In recent years, the importance of DPP-IV inhibition has grown beyond the treatment of type 2 diabetes. Recent evidence may indicate that DPP-IV inhibition may be of therapeutic significance in the field of cancer treatment [13,14]. Further research is focused on the potentially favorable cardiovascular effects of DPP-IV inhibition, as well as its effects on immune modulation [15,16,17,18]. A possible connection between DPP-IV inhibition and tackling neurological disorders has also been the subject of research attention [19,20]. However, in 2016, the FDA issued warnings about the increased risk of heart failure when using saxagliptin and alogliptin based on postmarketing studies and clinical trials [21,22,23,24]. The potential uses of DPP-IV inhibitors in treating conditions other than diabetes have been reviewed in the literature [25,26,27,28]. At present, there are twelve DPP-IV inhibitors that have been approved for use in clinical practice by the respective regulatory agencies, and there are many more in various stages of clinical trials [29]. In light of their various therapeutic uses, the development of novel and selective DPP-IV inhibitors remains an ongoing research challenge.

The prevailing classification of DPP-IV inhibitors divides them into two major classes—peptidomimetic and non-peptidomimetic. The peptidomimetic class of inhibitors has two groups—glycine- and β-alanine-based inhibitors. Both classes are designed based on natural DPP-IV substrates and bear a resemblance to them. The non-peptidomimetic series is structurally more diverse compared to the peptidomimetic inhibitor group [29,30]. Among the approved DPP-IV inhibitors, seven belong to the peptidomimetic group, and the remaining five are classified as non-peptidomimetic [29].

The present review aims to provide an overview of the synthetic methodologies that give rise to different heterocyclic DPP-IV inhibitors.

## 2. Five-Membered Heterocycles

### 2.1. Five-Membered Heterocycles with One Heteroatom and Their Benzo-Fused Derivatives

#### 2.1.1. Cyanopyrrolidine

**Figure 1:**
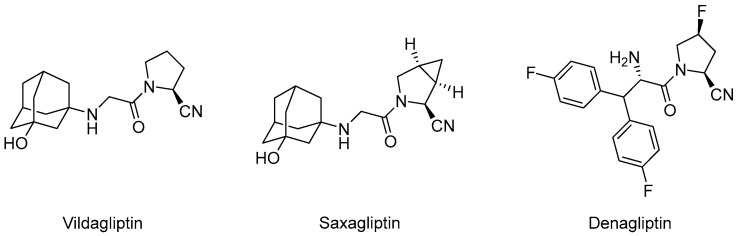
Cyanopyrrolidine-containing DPP-IV inhibitors.

Cyanopyrrolidines are among the most prominent and well-researched groups of DPP-IV inhibitors of the peptidomimetic group [31]. These compounds resemble the proline-containing DPP-IV substrate, and several approved drugs contain this fragment as well—Vildagliptin [32], saxagliptin [33], and Denagliptin [34] (Figure 1). The structure–activity relationship and the history of the development of the first inhibitors of this class have been reviewed in the literature [31,35].

**Scheme 1:**
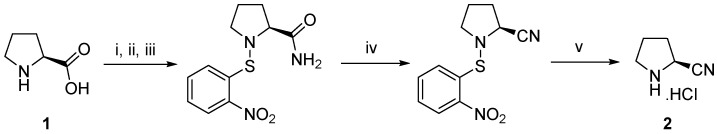
Reagents and conditions: (i) 2-Nitrobenzenesulfenyl chloride, 2 N NaOH; (ii) *N*-hydroxysuccinimide, carbodiimide; (iii) conc. NH_4_OH, dioxane, 94% (over three steps); (iv) imidazole, POCl_3_, pyridine, 95%; (v) 4 N HCl/dioxane, diethyl ether, 90%.

The methods of preparation usually use proline (**1**) as the starting material, which, upon conversion to amide and the subsequent dehydration, yields the target 2-cyanopyrrolidine (**2**). This approach was first described by Ashworth et al. [36] (Scheme 1).

**Scheme 2:**
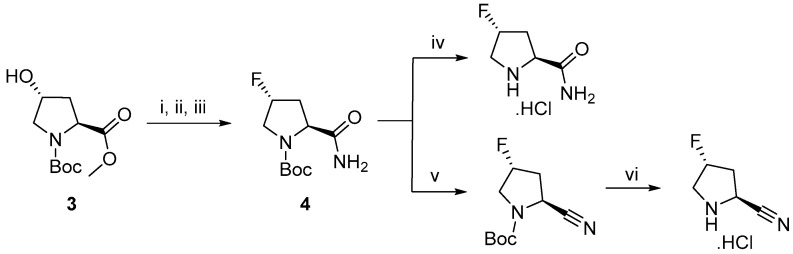
Reagents and conditions: (i) DAST, dry CH_2_Cl_2_, -78 °C to rt; (ii) 2 equiv. LiOH, AcCN-H_2_O (3-1), rt; (iii) EDC, HOBt, NH_3_, DMF-AcCN; (iv) 4 M HCl, ethyl acetate; (v) cyanuric chloride, DMF, quantitative yield; (vi) 2 M HCl, H_2_O-MeOH, quantitative yield.

Trifluoroacetic anhydride has also been widely utilized as a dehydrating agent for the conversion of amide to a nitrile group since the work of Villhauer et al. was published [37]. Further modification of the obtained 2-cyanopyrrolidine, especially its incorporation in peptides, can be achieved by the use of peptide coupling reagents [38]. The solid-phase synthesis of cyanopyrrolidine-containing peptides has also been reported [39]. This process starts with readily available Fmoc-proline being loaded on Rink-amide MBHA resin. The key dehydration reaction for obtaining the 2-cyanopyrrolidine fragment was again performed with trifluoroacetic anhydride [39]. Additional modifications of the pyrrolidine ring, such as the placement of an additional fluorine atom as a way to increase the potency of prospective inhibitors, have also been explored [40]. Fluorinated derivatives were obtained from the methyl ester of Boc-4-hydroxyproline **3** using diethylaminosulfur trifluoride (DAST) as a mild alcohol fluorinating agent [41,42]. The subsequent alkaline ester hydrolysis and coupling reaction with ammonia that were achieved with 1-[3-(dimethylamino)propyl]-3-ethylcarbodiimide hydrochloride (EDC) and 1-hydroxybenztriazole (HOBt) produced the fluorinated amide derivative **4** [43] (Scheme 2).

The coupling of the obtained pyrrolidine derivatives with amino acids, for example, aspartic and glutamic acid, has given rise to highly potent DPP-IV inhibitors [44,45].

#### 2.1.2. Indole and Isoindole

**Scheme 3:**
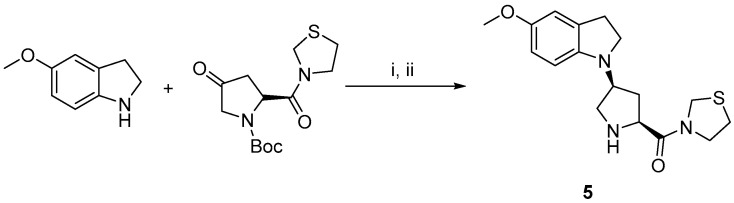
Reagents and conditions: (i) NaBH(OAc)_3_, AcOH, 93%; (ii) H^+^, 78%.

Inhibitors of DPP-IV do not typically contain indole derivatives. The very first indole-containing DPP-IV inhibitors were documented by Sakashita et al. [46]. The application of NaBH(OAc)_3_ in a reductive amination process yielded compound **5**, which has s 5-methoxyindoline moiety (Scheme 3).

**Scheme 4:**
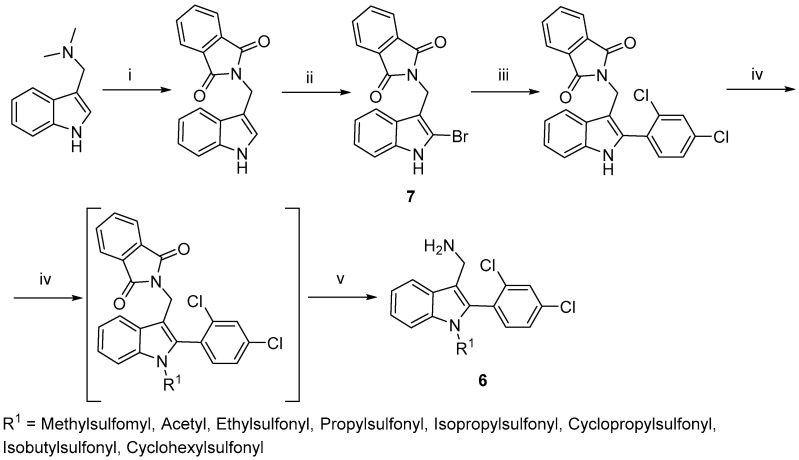
Reagents and conditions: (i) MeI, THF, potassium phthalimide, DMF, 150 °C, 5 h, 64%; (ii) THF, CHCl_3_, pyridinium tribromide, −10 °C, 3 h, 56%; (iii) 2,4-dichlorophenylboronic acid, Pd(PPh_3_)_4_, LiCl, Na_2_CO_3_, toluene, EtOH, 105 °C, 4 h, 30%; (iv) R_1_SO_2_Cl or R_1_COCl, NaH, DMF, 0 °C, 16 h 31–50%; (v) H_2_N-NH_2_, EtOH, rt for 1 h, 84–91%.

To develop novel compounds exhibiting enhanced selectivity for DPP-IV in comparison to DPP-9, a series of aryl-substituted indoles **6** and their sulfonamide derivatives were prepared [47]. The incorporation of the aryl group at C-2 was achieved through Suzuki coupling of **7** with aryl boronic acid, followed by the incorporation of an additional amino group introduced using potassium phthalimide in a Hoffman elimination reaction (Scheme 4).

**Scheme 5:**
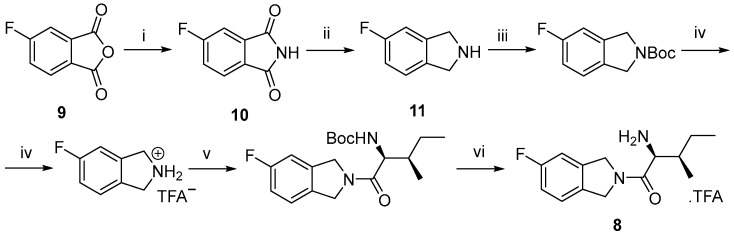
Reagents and conditions: (i) NH_4_OH, THF, thermal dehydration, 95–99%; (ii) borane, TFH; (iii) Et_3_N, Boc_2_O, CH_2_Cl_2_, 21–33% over two steps; (iv) TFA, CH_2_Cl_2_, 95–99%; (v) *allo*-Ile-Boc, TBTU, Et_3_N, CH_2_Cl_2_, 38–80%; (vi) TFA, CH_2_Cl_2_, 95–99%.

According to reports, isoindole derivatives can inhibit both DPP-IV and DPP-8/9 but with greater selectivity for DPP-8/9 [48]. A six-step synthetic route, starting with substituted phthalic anhydride **9** was used to prepare the lead compound **8** (Scheme 5). Key stages from the synthetic procedure include amidation and thermal ring closure to phthalimide derivative **10** and final reduction with borane to isoindole **11**. Peptide coupling chemistry was successfully used to incorporate the peptide side chain into the isoindole ring. Further research resulted in the introduction of a more complex peptide side chain, allowing higher selectivity for DPP-8 over DPP-9 [49].

### 2.2. Five-Membered Heterocycles with Two Heteroatom and Their Benzo-Fused Derivatives

#### 2.2.1. Pyrazole

**Figure 2:**
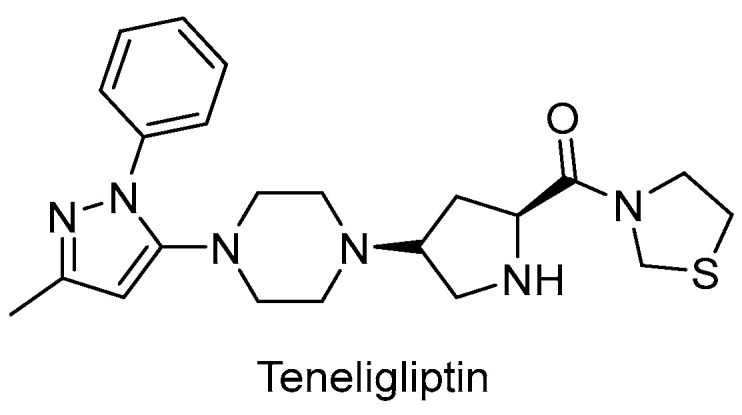
Structure of Teneligliptin.

A prominent example of pyrazole in the context of DPP-IV inhibition is the drug Teneligliptin (Figure 2), developed by Yoshida et al. and approved for clinical use in Japan [50].

**Scheme 6:**
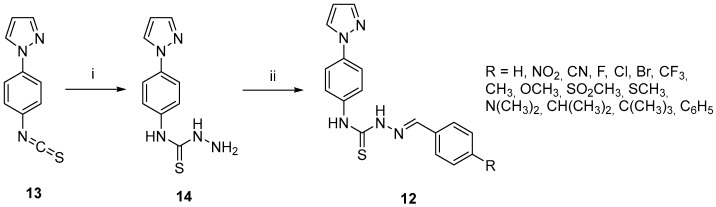
Reagents and conditions: (i) NH_2_NH_2_·H_2_O, ethanol, rt, 4 h; (ii) RC_6_H_4_CHO, ethanol, reflux, 8 h, 75–90%.

The inclusion of the pyrazole ring system in potential inhibitors significantly affects the anticipated interactions of the inhibitor and the DPP-IV active site, facilitating π-cation interactions with Arg358 and Tyr666, thereby engaging both S1 and S2 pockets of DPP-IV [29,51]. This research conducted by Server et al. on thiosemicarbazone DPP-IV inhibitors featuring a pyrazole ring **12** elucidates this conclusion appropriately (Scheme 6) [52]. The proposed inhibitors were obtained through a two-step synthetic protocol beginning with 4-(1*H*-pyrazol-1-yl)phenyl isothiocyanate (**13**), which, when treated with hydrazine hydrate, yielded the corresponding thiosemicarbazide product **14**. Condensation with substituted aromatic aldehydes produced the desired thiosemicarbazone functionality [53].

**Scheme 7:**
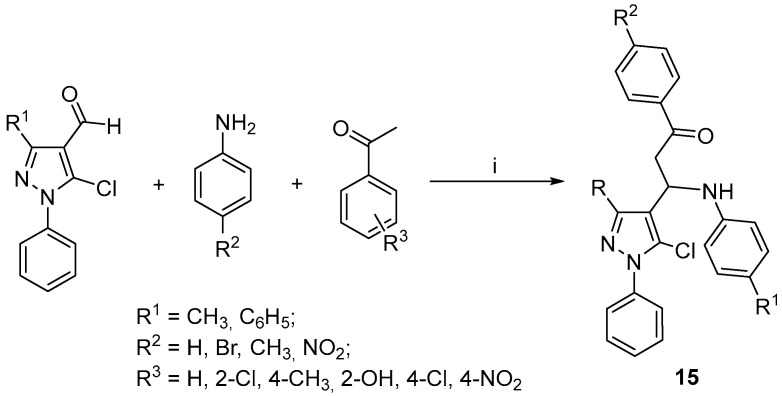
Reagents and conditions: (i) Bi(NO_3_)_2_, ethanol, 60–70 °C, 3–5 h, 75–96%.

A further instance of functionalizing 1-phenyl-1*H*-pyrazole derivatives in the development of DPP-IV inhibitors is the application of a one-pot Mannich reaction by Nidhar et al. [54]. The synthesized inhibitors **15** incorporated a β-amino acid side chain, which is crucial for enhancing affinity toward the DPP-IV enzyme (Scheme 7). The authors described 1-phenyl-1*H*-pyrazole as participating in important hydrophobic interactions with the amino acids Trp629, Ser630, Tyr631, and Tyr547. The synthesized inhibitors exhibited IC_50_ values in the nanomolar range, indicating their potential for further investigation. The authors also investigated the addition of a chalcone side chain [55].

**Scheme 8:**
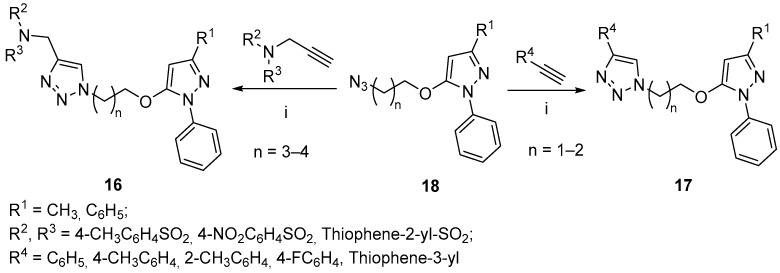
Reagents and conditions: (i) CuI/DCM and DIPEA and Me_6_TREN, 90–98%.

The authors also investigated the insertion of a persulfonimide side chain to the pyrazole heterocycle using a triazole linker **16**, **17** [56]. The addition of the persulfonimide fragment is expected to improve DPP-IV-inhibitory potential by interacting with Arg125, Gly741, and Trp629. Furthermore, the linker chain(1,2,3-triazole) can interact with His740, Val122, and Glu205. The two fragments were linked, and the triazole ring was synthesized using a click-chemistry approach based on a 1,3-dipolar cycloaddition reaction of alkynes and azide-substituted pyrazole **18** (Scheme 8).

**Scheme 9:**
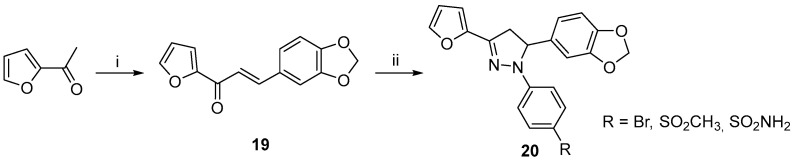
Reagents and conditions: (i) Piperonal, 10% NaOH, absolute ethanol, rt 24 h; (ii) 4-substituted phenylhydrazine hydrochloride, AcOH, absolute ethanol, reflux 10 h, 85–87%.

Another study described the use of the classical synthetic approach to construct pyrazole derivatives from α,β-unsaturated ketones **19** in reaction with phenylhydrazines, resulting in novel DPP-IV inhibitors featuring an additional furan ring **20** (Scheme 9) [57].

**Scheme 10:**
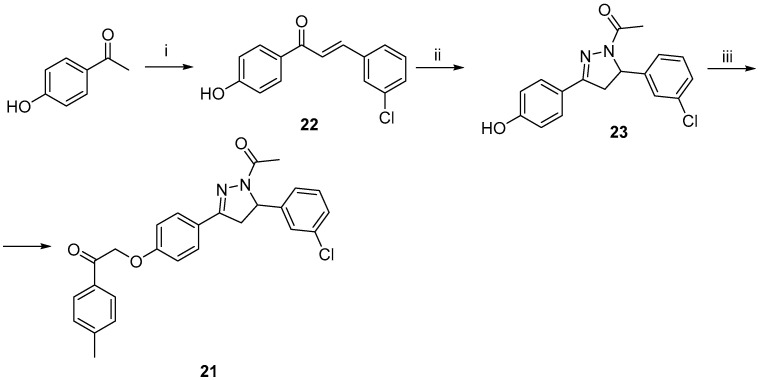
Reagents and conditions: (i) 3-chlorobenzaldehyde, NaOH, MeOH, reflux, 6 h; (ii) NH_2_NH_2_·H_2_O, AcOH, 90 °C, 81%; (iii) 2-bromo-1-(*p*-tolyl)ethan-1-one, dry acetone, K_2_CO_3_, reflux, 8–10 h, 61–89%.

**Scheme 11:**
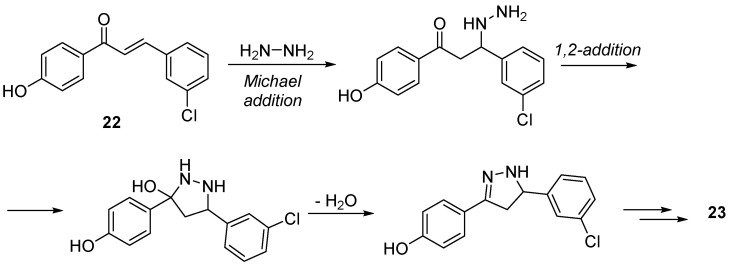
Mechanism of the formation of pyrazole-derivative **23**.

Sharma et al. employed a comparable method for constructing the target heterocycle in their research aimed at identifying novel potent DPP-IV inhibitors (Scheme 10) [58]. Their lead compound **21** was synthesized in a three-step procedure, with the key reaction between the substituted chalcone **22** and hydrazine in an acetic acid medium, resulting in the formation of the heterocycle **23** and achieving *N*-acylation in a single stage. This approach provides a versatile method for synthesizing pyrazole derivatives through the use of hydrazine and 1,3-difunctional compounds, including 1,3-dicarbonyl compounds, chalcones, β-ketoesters, etc. [59]. The reaction likely occurs through the initial conjugate addition of the hydrazine to the β-position of **22**, followed by ring closure via 1,2 addition to the carbonyl group and subsequent elimination of water (Scheme 11) [60]. The final Williamson reaction involving the substituted phenacetyl bromide derivative yielded the final product **21**. The obtained compound exhibited an IC_50_ within the micromolar range.

**Scheme 12:**
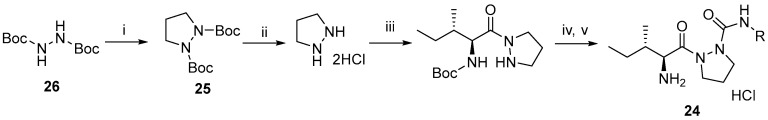
Reagents and conditions: (i) 50% NaOH, dibromopropane, Et_4_NBr, toluene, reflux, 5 h, 87%; (ii) 4 M HCl, dioxane, 100%; (iii) Boc–isoleucine, EDCI, TEA, CH_2_Cl_2_, 78%; (iv) RNCO, CH_2_Cl_2_, rt, 12 h, 70–80%; (v) 4 M HCl, dioxane–EtOAc, rt, 12 h, 87%.

The pyrazolidine scaffold is present in potential DPP-IV inhibitors, in particular when coupled with isoleucine **24** (Scheme 12) [61]. These inhibitors were derived from previously reported cyano-pyrazolidines, which demonstrated inhibitory activity in the micromolar range [62]. The core heterocycle **25** was synthesized through a reaction between Boc-protected hydrazine **26** and 1,3-dibromopropane in the presence of Et_4_NBr.

#### 2.2.2. Isoxazole

**Scheme 13:**
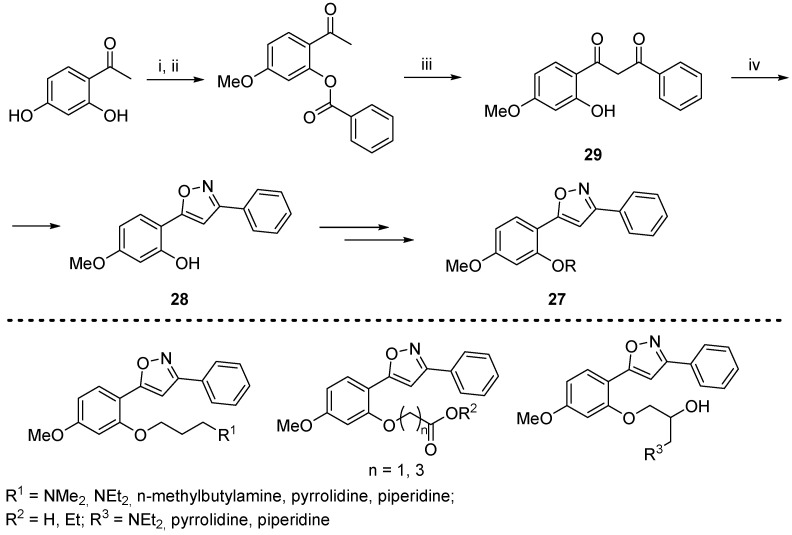
Reagents and conditions: (i) (CH_3_)_2_SO_4_, K_2_CO_3_, acetone; (ii) benzoyl chloride, pyridine, 1 h, rt, 82% over two steps; (iii) KOH, pyridine, 8 h, rt, 82%; (iv) NH_2_OH·HCl, pyridine, 45%.

The first documented isoxazole-based DPP-IV inhibitors were developed in work by Kumar et al., which included the synthesis of a series of 3,5-diarylisoxazoles **27** [63]. The key reaction used a classical approach to construct the heterocycle **28** through the interaction of 1,3-dicarbonyl compound **29** and hydroxylamine (Scheme 13). The intermediate **28** was obtained in moderate yield, which is common when unsymmetrical diketones undergo this process [64]. The final target products **27** are synthesized via *O*-alkylation of the phenolic hydroxyl group.

**Scheme 14:**
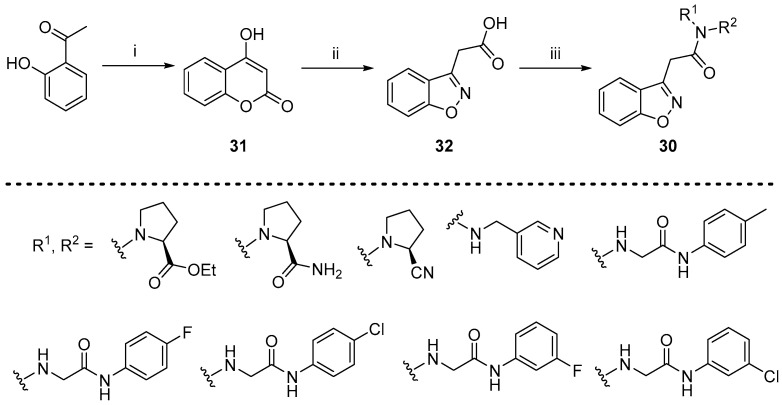
Reagents and conditions: (i) Na metal, 70–80 °C, 1 h, 45%; (ii) NH_2_OH.HCl, NaHCO_3_, MeOH, 64–66 °C, 15 h, 40%; (iii) NR^1^R^2^.TFA/NR^1^R^2^, EDCl, HOBt, TEA, DCM, rt, 16 h, 32–71%.

A recent study by Karandikar et al. examined benzo-fused isoxazoles with an acetamide side chain **30** [65]. The compounds were synthesized through a three-step synthetic strategy, with the key reaction being a Posner ring transformation of 4-hydroxycoumarin **31** to form 1,2-benzisoxazol-3-acetic acid **32** (Scheme 14). The amide side chain was introduced using coupling reagents. The introduction of an additional glycine fragment in the amide side chain, serving as a spacer, improved the DPP-IV-inhibitory activity.

#### 2.2.3. Imidazole

**Scheme 15:**
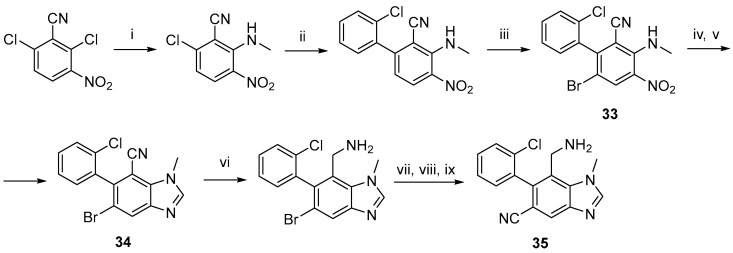
Reagents and conditions: (i) CH_3_NH_2_, THF, 82%; (ii) *o*-ClC_6_H_4_-B(OH)_2_, Pd_2_dba_3_, DavePhos, K_3_PO_4_, DMA, 68 °C, 74%; (iii) Br_2_, AcOH, 88 °C, 94%; (iv) Fe, AcOH; (v) formic acid, polyphosphoric acid, 73% over two steps; (vi) BH_3_-THF, reflux then HCl, 33%; (vii) Boc_2_O; (viii) Zn(CN)_2_, Pd_2_dba_3_, DavePhos, K_3_PO_4_, DMA, 92 °C; (ix) TFA, DCM, 70% over three steps.

The first documented DPP-IV inhibitors with benzimidazole as a core structure were developed in 2008 by Wallace et al. [66]. The molecular design of the prospective inhibitors was based on available crystallographic data of DPP-IV in conjunction with 2-phenylbenzylamine. The synthetic methodology consists of several steps, with the key ring-forming process being the condensation of substituted *o*-diamine, formed in situ from **33** with formic acid. Following the cyanide reduction of **34**, amine Boc protection, Pd-coupling reaction to introduce the CN group, and final deprotection, the target product **35** was obtained (Scheme 15). The authors described the versatility of the synthetic protocol, enabling the synthesis of molecules with diverse substituents to the heterocycle.

**Scheme 16:**
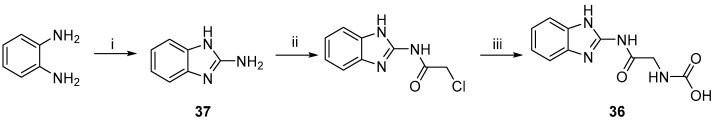
Reagents and conditions: (i) BrCN, H_2_O, 4 h, 70%; (ii) chloroacetyl chloride, dry benzene, 0–5 °C, then reflux for 6 h, 85%; (iii) aminoacetic acid, pyridine, EtOH, 6 h, 75%.

More recently, Sunil et al. demonstrated that aminosubstituted benzimidazoles **36** could be a novel class of potent DPP-IV inhibitors [67]. The parent 2-amino benzimidazole **37** was prepared by treating *o*-phenylenediamine with cyanogen bromide (Scheme 16).

**Scheme 17:**
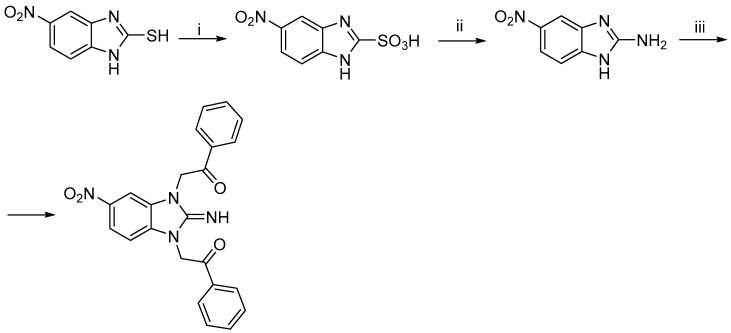
Reagents and conditions: (i) KMnO_4_, NaOH, H_2_O, reflux, 52%; (ii) NH_4_OH, 150 °C in welded ampoule, 77%; (iii) Tetrabutylammonium bromide, dry K_2_CO_3_, 2-chloro-1-phenylethan-1-one, 25 °C, acetonitrile, 82%.

Katarina Tomovic et al. recently reported another example of *N*-substituted benzimidazoles as potential DPP-IV inhibitors [68]. The authors obtained two series of benzimidazole derivatives starting with commercially available substituted 2-mercapto-benzimidazoles (Scheme 17).

### 2.3. Five-Membered Heterocycles with Three Heteroatoms and Their Benzo-Fused Derivatives

#### 2.3.1. Triazole

**Scheme 18:**
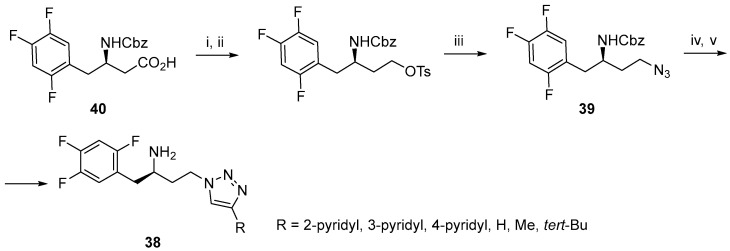
Reagents and conditions: (i) NaBH_4_, I_2_, THF, 88%; (ii) TsCl, TEA, DCM, 76%; (iii) NaN_3_, DMSO, 80 °C, 85%; (iv) alkynes, Cu(OAc)_2_, CH_3_CN, (v) 10% Pd/C-H_2_, MeOH, 65–85%.

Apart from triazoles fused to larger heterocycles, which are reviewed in Section 4, triazole-based DPP-IV inhibitors can be found in early 2016 in the literature. Gundetti et al. reported the synthesis and DPP-IV-inhibitory activity of 1,2,3-tiazoles **38** designed as analogues of Sitagliptin [69]. The suggested synthetic protocol is based on click chemistry—1,3-dipolar cycloaddition between azide **39** and appropriate alkyne (Scheme 18). Starting with (3*R*)-*N*-(tert-butoxycarbonyl)-3-amino-4-(2,4,5-trifluorophenyl)butanoic acid **40**, the target compounds are obtained in a total of five synthetic steps and showed nanomolar IC_50_ values.

**Scheme 19:**
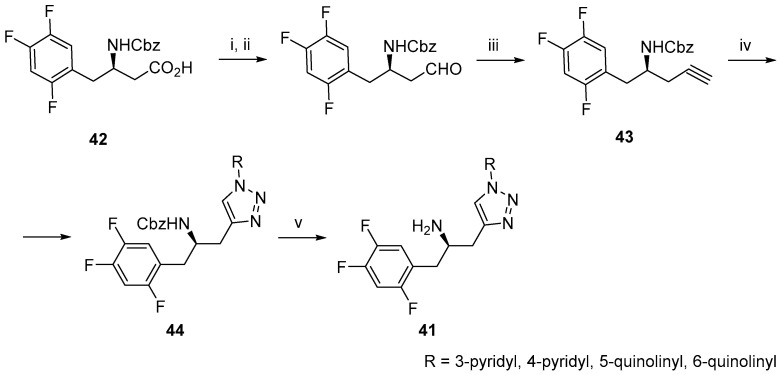
Reagents and conditions: (i) CAN/MeOH, rt, 5 h, 97%; (ii) DIBAL, −78 °C, 3 h, 86%; (iii) Bestmann–Ohira reagent, K_2_CO_3_, MeOH, rt, 14 h, 94%; (iv) arylazides, Cu(OAc)_2_, CH_3_CN, rt, 3 h, 98–82%; (v) HBr/AcOH, 80 °C, 14 h, 65–85%.

The authors extended the scope of the synthetic methodology to the construction of analogues with bulkier groups **41** attached to the heterocycle [70]. The starting **42** was converted to a terminal alkyne **43** in three steps with the use of the Ohira–Bestmann reagent; subsequent azide cycloaddition yielded the final heterocycle **44** (Scheme 19). The obtained novel inhibitors performed poorly in comparison with the previous series; however, they still showed micromolar range IC_50_ values. The procedure was further used by the authors to install bulkier substituents to the triazole heterocycle [71].

**Scheme 20:**
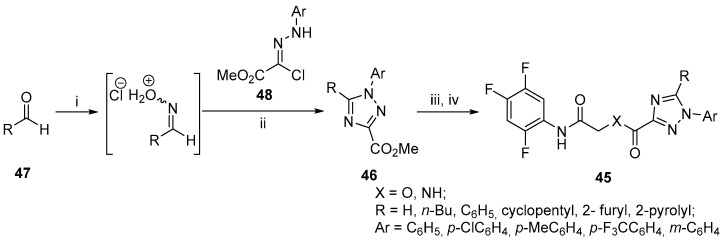
Reagents and conditions: (i) NH_2_OH.HCl, toluene; (ii) toluene, reflux, 1–2 h, 28–91%, (iii) DBU, MeOH, r.t., 6 h, >90%; (iv) 2-hydroxy-*N*-(2,4,5- trifluorophenyl)acetamide or 2-amino-*N*-(2,4,5-trifluorophenyl)acetamide, Bis(2-oxo-3-oxazolidinyl)phosphinic chloride (BOP-Cl), NEt_3_, CH_2_Cl_2_, 4 h, 71–92%.

Sitagliptin and Vildagliptin served as important references in the development of novel 1,2,4-triazole derivatives **45** as DPP-IV inhibitors [72]. The suggested inhibitors exhibited IC_50_ values in the nanomolar range as well as excellent selectivity for DPP-IV over Quiescent cell proline dipeptidase (QPP) and DPP-8/9. The triazole **46** was again constructed via 1,3-dipolar cycloaddition from aldehydes **47** with hydrazonoyl hydrochlorides **48** in the presence of hydroxylamine hydrochloride (Scheme 20) [73]. The process demonstrates a high functional group tolerance and was subsequently adapted by the authors to be able to use nitriles as starting reagents as well [74]. Another variation is the use of Vilsmeier reagent with hydrazonoyl hydrochlorides [75].

**Scheme 21:**
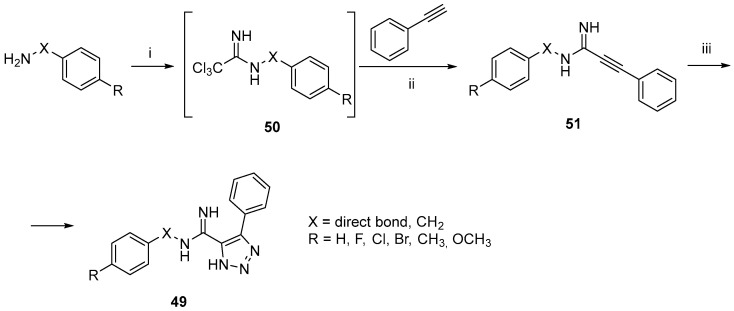
Reagents and conditions: (i) acetonitrile, rt, 10 min; (ii) Et_3_N, CuI, rt, 5 h; (iii) NaN_3_, methanol-water, rt, 8 h, 60–77% overall yield.

The reaction of substituted alkynes with sodium azide as a route to 1,2,3-triazole derivatives **49** containing carboximidamide functionality has also been documented [76]. The compounds demonstrated remarkable DPP-IV-inhibitory activity, exhibiting IC_50_ values in the nanomolar range. The synthesis consists of three steps, in which the required trichloroacetimidamide **50** is generated in situ and reacted with phenylacetylene to yield the appropriately substituted alkyne **51** (Scheme 21). The final reaction of the obtained **51** with sodium azide occurs at room temperature in the water–methanol medium.

#### 2.3.2. Oxadiazole

**Scheme 22:**
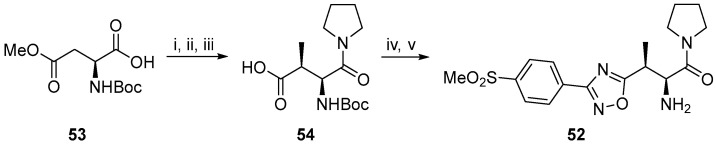
Reagents and conditions: (i) EDC, HOBt, DIEA, pyrrolidine, DMF; (ii) KHMDS, MeI, −78 °C; (iii) LiOH, THF, H_2_O; (iv) CDI, 4- methanesulfonylbenzamidoxime, rt, 1 h, then 110 °C, 12 h; (v) TFA/CH_2_Cl_2_, 1 h.

A good example of 1,2,4-oxadiazole-based DPP-IV inhibitors can be found in the work of Xu et al. [77]. A series of highly potent and selective DPP-IV inhibitors **52** were prepared by the authors and are recognized as some of the most powerful inhibitors in the literature. The preparation of **52** involves a coupling reaction between aspartic acid derivatives **53** and substituted benzamidoxime, mediated by 1,1-carbonyl diimidazole (CDI) (Scheme 22). The synthetic route consists of five steps, beginning with the Boc-protected methyl ester of aspartic acid **53**. The subsequent coupling reaction of **53** introduces a pyrrolidine functionality, followed by enolate alkylation at the α-position in the resulting **54**.

**Scheme 23:**
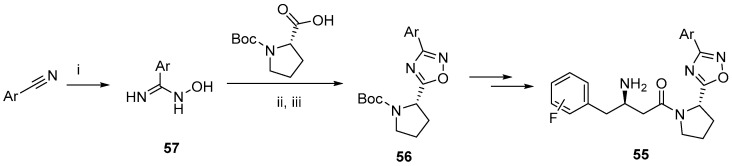
Reagents and conditions: (i) NH_2_OH, K_2_CO_3_, MeOH; (ii) DIC, DCM; (iii) pyridine, reflux.

Nordhoff et al. also observed the increased potency of 1,2,4-oxadiazole-based DPP-IV inhibitors that contain a pyrrolidine moiety **55** [78]. The authors investigated the inhibitory activity of 1,3,4-oxadiazole derivatives as DPP-IV inhibitors, but they were outperformed by their 1,2,4- analogues **55**. The key ring-forming reaction to obtain **56** was a condensation of amidoxime derivatives **57** with carboxylic acids (Scheme 23).

## 3. Six-Membered Heterocycles

### 3.1. Six-Membered Heterocycles with One Heteroatom and Their Benzo-Fused Derivatives

#### 3.1.1. Pyridine, Piperidine, Quinoline, and Isoquinoline

**Scheme 24:**
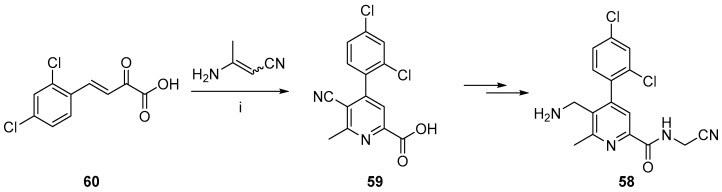
Reagents and conditions: (i) *t*-BuOK, AcCN, rt, 16 h, 81%.

The work of Kaczanowska et al. outlines aminomethyl pyridine derivatives **58** as nanomolar DPP-IV inhibitors [79]. The synthesis of the core heterocycle **59** was based on earlier research by the authors [80]. The process can be described as a base-catalyzed cyclocondensation between unsaturated oxo acid **60** and β-aminocrotonitrile, formed in situ during the course of the reaction (Scheme 24).

**Scheme 25:**
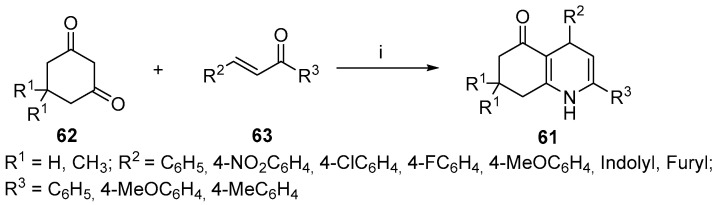
Reagents and conditions: (i) NH_4_OAc; 10 mol% *p*-toluenesulfonic acid, MeOH, reflux, 75–93%.

Kumar et al. explored the antihyperglycemic properties of partially saturated quinoline derivatives and evaluated the compounds against several in vitro diabetes models [81]. The authors prepared 2,4-diaryl substituted polyhydroquinolines **61** using a reaction between cyclic 1,3-diketones **62** and chalcones **63** in the presence of ammonium acetate and *p*-toluenesulfonic acid as a catalyst (Scheme 25). The synthetic protocol was referenced from the literature [82].

**Scheme 26:**
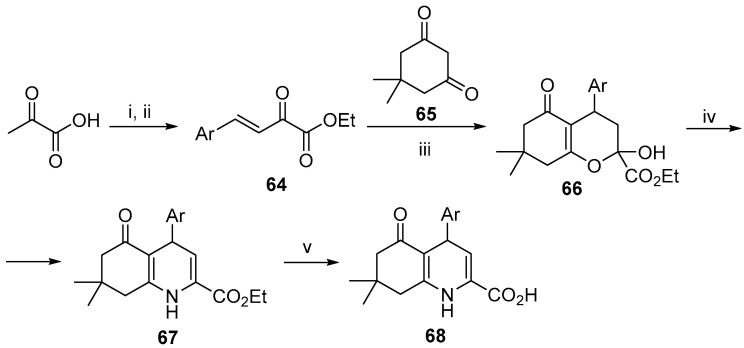
Reagents and conditions: (i) KOH, MeOH, rt, 10–15 h; (ii) CH_3_COCl, EtOH, 70 °C, 6–8 h, 85–93% over two steps; (iii) *p*-toluenesulfonic acid, MeOH, rt, 78–92%; (iv) NH_4_OAc, EtOH, reflux, 88–95%; (v) NaOH, MeOH, 80–97%.

In order to obtain polyhydroquinolines with an ester functionality, the authors employed β,γ-unsaturated α-ketoesters **64**. This was achieved through a base-catalyzed reaction between aromatic aldehydes and pyruvic acid, followed by an esterification (Scheme 25) [81]. Subsequent ring-forming reaction with dimedone **65** yielded the chromene derivative **66**, which was transformed into the target quinoline derivative **67**. The products **67** were transformed into the corresponding carboxylic acids **68** (Scheme 26).

**Scheme 27:**
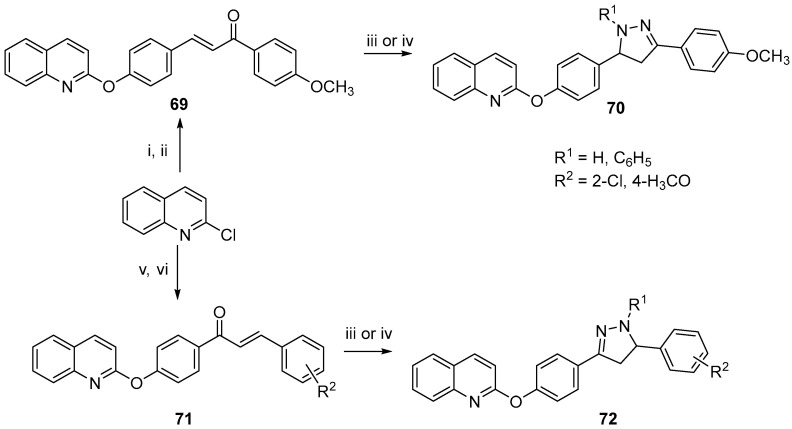
Reagents and conditions: (i) 4-hydroxybenzaldehyde, DMF, K_2_CO_3_, 100 °C 6 h; (ii) 4-methoxyacetophenone, ethanol, 10% aq. NaOH, stirring for 2 h, 64% over two steps; (iii) hydrazine hydrate, ethanol, reflux 4 h, 80%; (iv) phenylhydrazine ethanol, reflux for 6 h, 75%; (v) 4-hydroxyacetophenone, DMF, K_2_CO_3_, reflux for 12 h; (vi) aldehydes (2-chlorobenzaldehyde or 4-methoxybenzaldehyde), ethanol, 10% aq. NaOH, stirring for 2 h, 57–65% over two steps.

Prospective quinoline inhibitors that contain a pyrazole substituent have also been explored [83]. The synthetic pathway starts from commercially available 2-chloroquinoline, which undergoes a reaction with 4-hydroxybenzaldehyde and subsequently with 4-methoxyacetophenone to yield the chalcone derivative **69** (Scheme 27). The cyclocondensation of **69** with hydrazine hydrate or phenylhydrazine gave the final products **70**. In another approach, 2-chloroquinoline reacted with 4-hydroxyacetophenone, leading to a product that was condensated with substituted aromatic aldehydes to furnish **71**. In a similar matter, the formation of the pyrazole ring was accomplished through the reaction of **71** with hydrazine hydrate or phenylhydrazine, and the target **72** was obtained.

#### 3.1.2. Coumarin

**Scheme 28:**
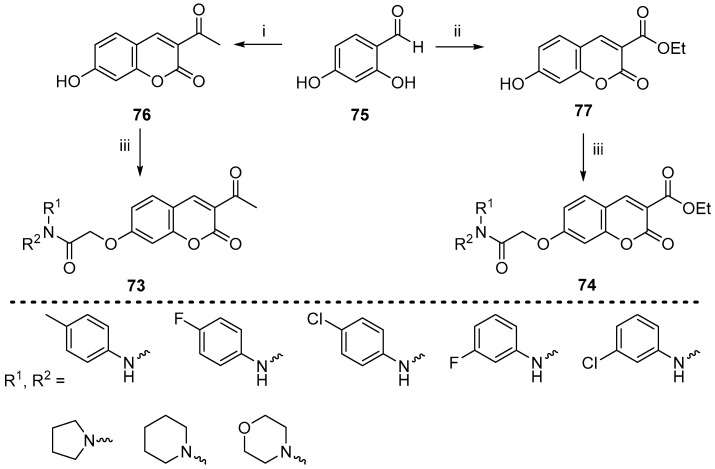
Reagents and conditions: (i) ethyl acetoacetate, piperidine, 0 °C to rt, overnight; (ii) KSF montmorillonite, 160 °C, 46%; (iii) substituted amine, anhydrous K_2_CO_3_, KI, DMF, 70–80 °C, 12–18 h, 43–91%.

The pioneering research on coumarin derivatives for the inhibition of DPP-IV can be attributed to the work of Soni et al. in 2016 [84]. The authors prepared a series of coumarins featuring an amide side chain **73** and **74** (Scheme 28). The compounds were synthesized using a two-step synthetic route, beginning with the condensation of 2,4-dihydroxybenzaldehyde **75** with acetoacetic or malonic ester to form the core coumarin heterocycle **76** and **77** [85,86]. The final products **73** and **74** were obtained by alkylation of the 7-hydroxyl group in **76** and **77**.

**Scheme 29:**
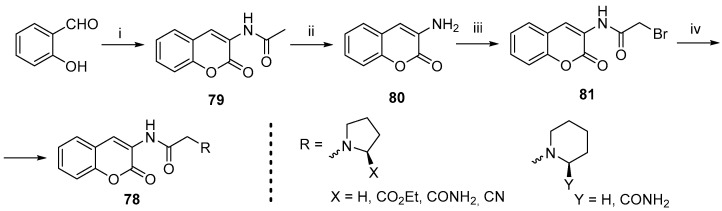
Reagents and conditions: (i) *N*-acetyl glycine, Ac_2_O, NaOAc, reflux, 7 h, 47%; (ii) EtOH, conc. HCl, reflux, 1 h, 61%; (iii) 2-bromoacetyl bromide, TEA, DCM, rt, 2 h, 89%; (iv) substituted amine, Et_3_N, DMF, rt, 16 h, 49–85%.

**Scheme 30:**
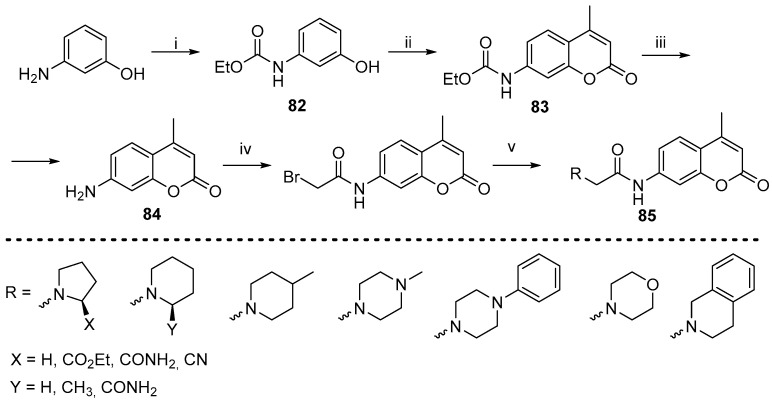
Reagents and conditions: (i) ethyl chloroformate, EtOAc, rt, 2 h, 48%; (ii) ethyl acetoacetate, 70% EtOH in H_2_SO_4_, rt, 16 h, 85%; (iii) AcOH, H_2_SO_4_, 106–108 °C, 1 h, 71% (iv) bromo acetyl bromide, Et_3_N, DCM, rt, 2 h, 93%; (v) substituted amine derivatives, TEA, DMF, rt, 16 h, 47–72%.

In subsequent research, the authors successfully synthesized a new series of amino-substituted coumarin derivatives [87]. Two novel series of coumarins bearing amine substituent on positions 3- and 7- were obtained. The synthesis of 3-aminocoumarin derivatives **78** was carried out using a two-step approach (Scheme 29). The initial 2-hydroxybenzaldehyde underwent a reaction with *N*-acetyl glycine in a Perkin reaction, resulting in the formation of the acetamide derivative **79**. Acidic deprotection yielded the 3-aminocoumarin as a free base **80** [88]. Acylation with bromoacetyl bromide furnished the substituted **81**, which was reacted with various amines to yield the derivatives **78**. The 7-aminocoumarin series was synthesized via a Pechmann condensation starting with substituted 3-aminophenol derivative **82** (Scheme 30). The resulting coumarin **83** was converted to its free base **84** and, similarly to the synthesis of the previous series, was alkylated with bromoacetyl bromide and reacted with amine derivatives to yield the final product **85**.

The authors employed a similar synthetic procedure to obtain a series of 7-aminocoumarin derivatives featuring a proline and sulfonamide moiety in the side chain [89].

**Scheme 31:**
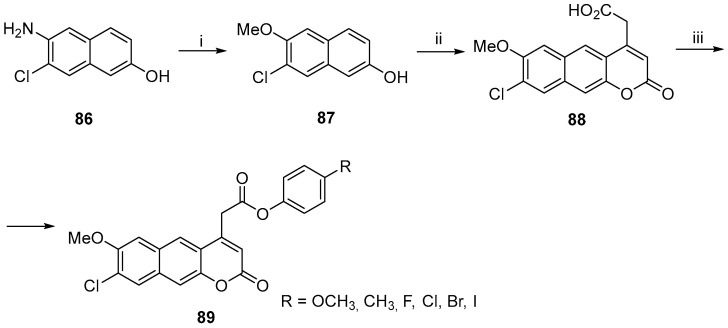
Reagents and conditions: (i) NaNO_2_, MeOH, AcOH, 6 h, rt, 43%; (ii) 3-oxoglutaric acid, conc. H_2_SO_4_, sonication, 1.5 h, 30 °C, 88%; or 3-oxoglutaric acid, conc. H_2_SO_4_, 20 h, rt, 80%; (iii) substituted phenol derivative, SOCl_2_, 63–76%.

The Pechmann condensation was also used in the recent research by Jasim et al. for the synthesis of coumarins, which incorporated an additional condensated benzene ring (Scheme 31) [90]. The synthetic protocol begins with substituted naphthalene **86**, which, upon diazotation and treatment with methanol, is transformed to its methoxy counterpart **87**. The Pechmann condensation of **87** with 3-oxoglutaric acid yielded the coumarin derivative **88**. The final products **89** were obtained after esterification of the carboxylic group with substituted phenols. In later studies, the authors replaced the methoxy group in **89** with chlorine, therefore enhancing the potency of the obtained compounds [91].

### 3.2. Six-Membered Heterocycles with Two Heteroatoms and Their Benzo-Fused Derivatives

#### 3.2.1. Piperazine

**Scheme 32:**
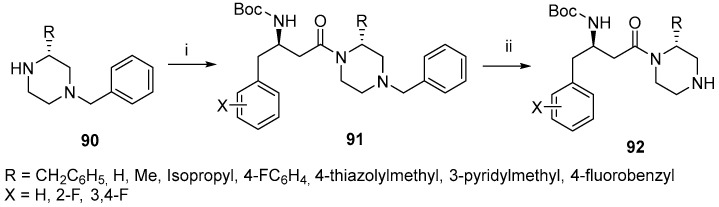
Reagents and conditions: (i) (*R*)-ArCH_2_CH(NHBoc)CH_2_CO_2_H, EDC, HOBt, DIEA, DMF; (ii) H_2_, Pd(OH)_2_, MeOH.

Substituted aryl-piperazines containing β-amino acids have been reported as nanomolar DPP-IV inhibitors [92]. The synthetic procedure begins with *N*-benzyl substituted piperazine **90**, which undergoes a coupling reaction with Boc-protected β-aminoacids to furnish **91**. After benzyl deprotection, the key intermediate **92** is produced. Subsequent alkylation on the unsubstituted nitrogen in **92** led to the formation of four groups of DPP-IV inhibitors (Scheme 32).

**Scheme 33:**
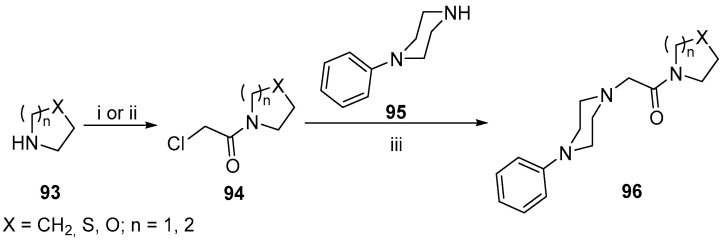
Reagents and conditions: (i) chloroacetyl chloride, TEA, DCM, −15 °C to 0 °C, 2 h, 80–93%; (ii) chloroacetic acid, DCC, DCM, 0 °C, 4 h; (iii) TEA, TFH, rt, 6–8 h, 64–87%.

Kushwaha et al. synthesized piperazines containing an additional heterocyclic substituent such as pyrrolidine, thiazolidine, piperidine, or morpholine (Scheme 33) [93]. The compounds were obtained from the respective five- or six-membered heterocycle **93**, which was treated with chloroacetyl chloride to give the intermediate **94**. The final substitution reaction with aryl-piperazine **95** gave the final products **96**.

**Scheme 34:**
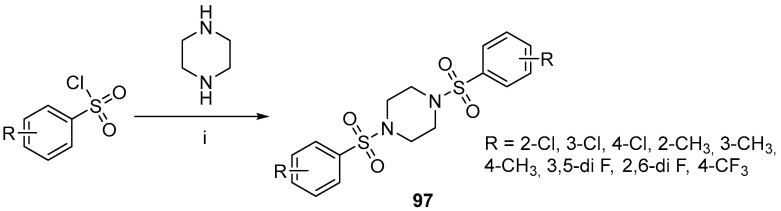
Reagents and conditions: (i) NaOH, acetone, reflux, 1.5 h, 1–76%.

Piperazine can be functionalized with sulfonamide moieties at the two nitrogen atoms, giving rise to sulfonamide-piperazine inhibitors **97** (Scheme 34) [94].

#### 3.2.2. Pyridazine

**Scheme 35:**
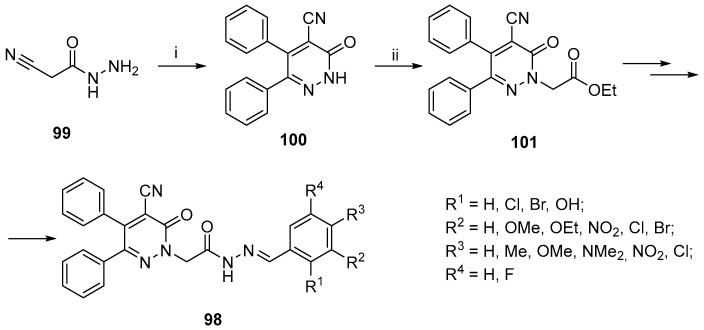
Reagents and conditions: (i) benzil, K_2_CO_3_, MeOH, reflux, 90–95%; (ii) ethyl 2-chloroacetate, K_2_CO_3_, anhydrous DMF, 2 h, good to excellent yield.

A recent report by Nidhar et al. showed the synthesis of novel pyridazine-acetohydrazide hybrids **98** and their DPP-IV-inhibitory activity in nanomolar concentrations [95]. The heterocycle was formed by a base-catalyzed reaction of 2-cyanoacetohydrazide (**99**) and benzil (Scheme 35). The obtained core heterocycle **100** was acylated, and the resulting product **101** was further converted to the target **98**.

#### 3.2.3. Quinazoline and Quinoxaline

**Figure 3:**
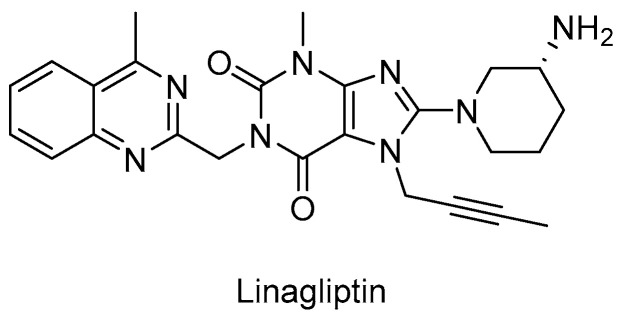
Structure of Linagliptin.

The quinazoline heterocycle could be seen in the approved drug Linagliptin (Figure 3), which is a highly potent DPP-IV inhibitor approved by the FDA in 2011 [96].

**Scheme 36:**
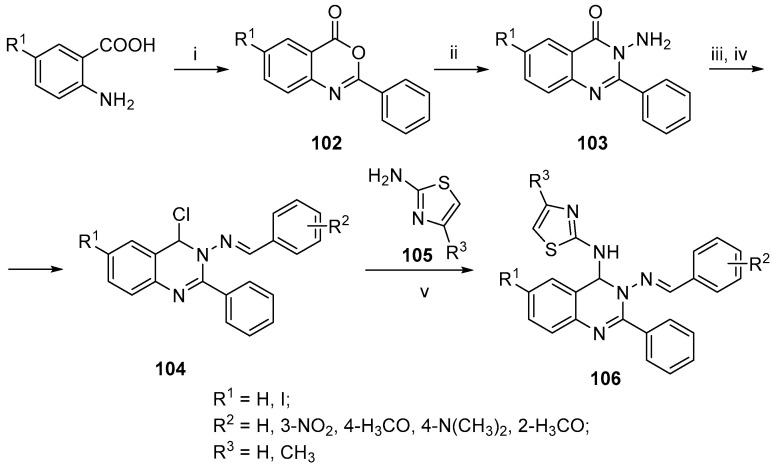
Reagents and conditions: (i) anhydrous pyridine, 2–8 °C, sodium bicarbonate, 1 h, 72–76%; (ii) H_2_NN_2_H.H_2_O, ethanol, 6 h, 68–74%; (iii) aldehydes, ethanol, glacial acetic acid, reflux, 8 h, 66–76%; (iv) anhydrous toluene, DIPEA, POCl_3_, reflux, 4–6 h, 64–82%; (v) phenol, reflux, 4 h, 70–78%.

Quinazoline derivatives coupled with thiazole have been identified as a novel class of DPP-IV inhibitors that also possess inhibitory potential against DPPH [97]. These molecules were synthesized in five steps, starting with anthranilic acid or its iodo-derivative (Scheme 36). The reaction with benzoyl chloride gave the benzoxazine derivative **102**, which reacted with hydrazine monohydrate to form the target quinazoline core **103**. Condensation with aldehydes and subsequent treatment with POCl_3_ resulted in the formation of the intermediate product **104**, which, when coupled with thiazole derivatives **105**, gave the final products **106**.

The authors also synthesized quinazolines bearing a thiazole ring at C-2 using the same methodology [98]. The obtained compounds showed IC_50_ values in the nanomolar range as well as good selectivity for DPP-IV over DPP-8/9. A similar synthetic approach could be found in the work of Zayed et al., describing novel quinazolinone derivatives featuring a sulfonamide side chain [99].

**Scheme 37:**
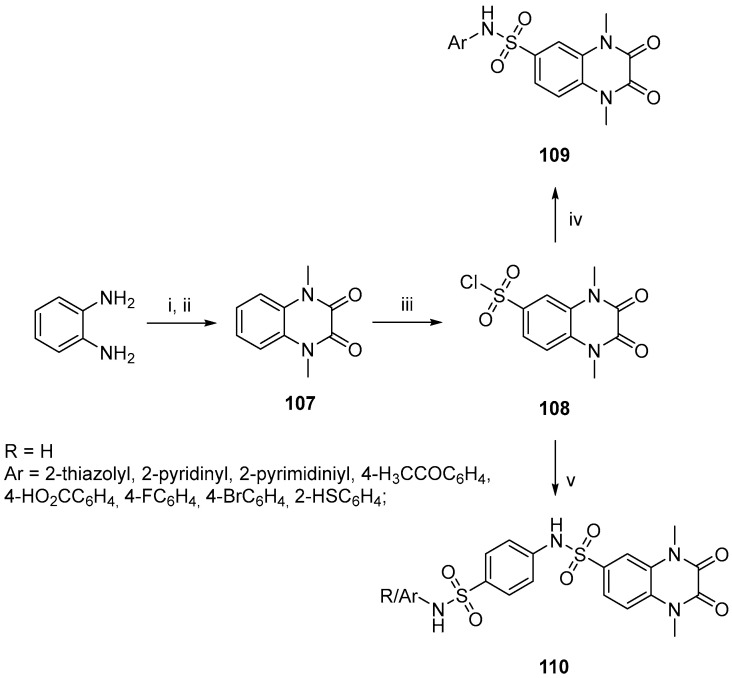
Reagents and conditions: (i) oxalic acid, 4 N HCl, reflux, 4 h; (ii) anhydrous K_2_CO_3_, DMF, methyl iodide, heating, 6 h, 99%; (iii) ClSO_3_H, heating, 90 °C, 3 h; (iv) heterocycles or primary amines, EtOH, reflux, 3 h, 72–75%; (v) sulfa drugs, Et_3_N, EtOH, 6 h, 81–85%.

Quinoxaline derivatives have also been reported as exhibiting DPP-IV-inhibitory activity and hypoglycemic properties (Scheme 37) [100]. The heterocyclic core **107** was obtained using a base-catalyzed condensation between *o*-phenylenediamine and oxalic acid, followed by alkylation with methyl iodide. In order to install a sulfonamide functionality, **107** was treated with chlorosulfonic acid to obtain the 6-sulfonyl chloride intermediate **108**. After a reaction of **108** with aromatic and heterocyclic amines, the products **109** were obtained. Alternatively, **108** reacted with sulfur-containing drugs such as sulfanilamide, sulfathiazole, sulfapyridine, and sulfadiazine to afford the derivatives **110**.

#### 3.2.4. Pyrimidine

**Figure 4:**
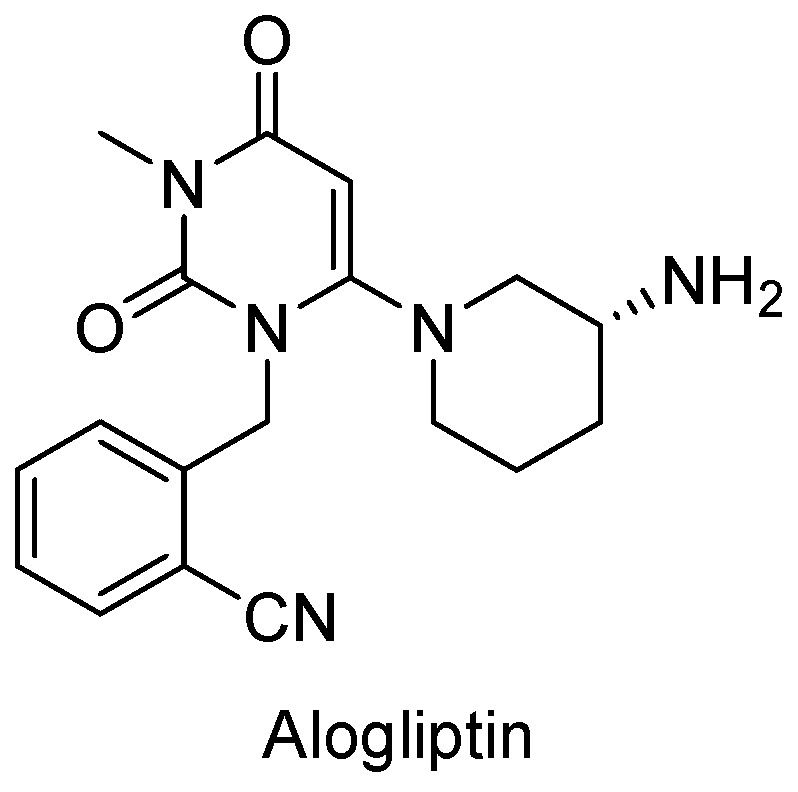
Structure of alogliptin.

The drug alogliptin (Figure 4) can be viewed as an example of a pyrimidine derivative as a DPP-IV inhibitor [101,102].

**Scheme 38:**
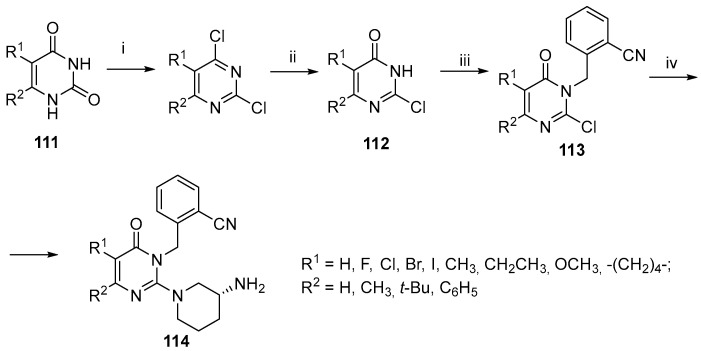
Reagents and conditions: (i) POCl_3_, dimethylaniline, reflux; (ii) NaOH, 47–76%; (iii) NaH, LiBr, 2-cyanobenzyl bromide, 20–52%; (iv) 3-(*R*)-aminopiperidine, NaHCO_3_, 60 °C, 49–74%.

Zhang et al. synthesized pyrimidinone derivatives from pyrimidinediones **111**, which underwent chlorination with POCl_3_, followed by selective alkaline hydrolysis to yield **112** [103]. Alkylation with cyanobenzyl bromide produced a mixture of regioisomers, of which **113** was further used by the authors to synthesize the target inhibitors **114** (Scheme 38).

**Scheme 39:**
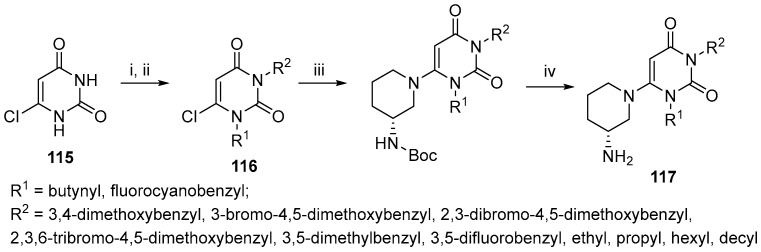
Reagents and conditions: (i) R^1^Br, DIPEA, DMF, rt; (ii) R^2^Br, K_2_CO_3_, KI, DMF, rt; (iii) (*R*)-3-(Boc-Amino)piperidine, K_2_CO_3_, DMF, 90 °C, N_2_ atmosphere; (iv) CH_2_Cl_2_, TFA, rt, 65–84%.

Further research on the pyrimidinone derivatives that incorporate an additional 3-aminopiperidine fragment can be seen in the work of Ning Li et al. [104]. The synthesis follows a four-step procedure starting with 6-chlorouracil (**115**), which undergoes alkylation on both nitrogen atoms to form the precursor **116**. After reacting **116** with (*R*)-3-(Boc-amino) piperidine and subsequent amine deprotection, the final products **117** were obtained (Scheme 39). A similar synthetic approach was employed by Vibhu Jha et al. in their research on novel *N*-methylated and *N*-benzylated pyrimidinediones [105]. Modification of the heterocyclic core **117** to incorporate benzoic acid moiety has also been explored [106,107].

**Scheme 40:**
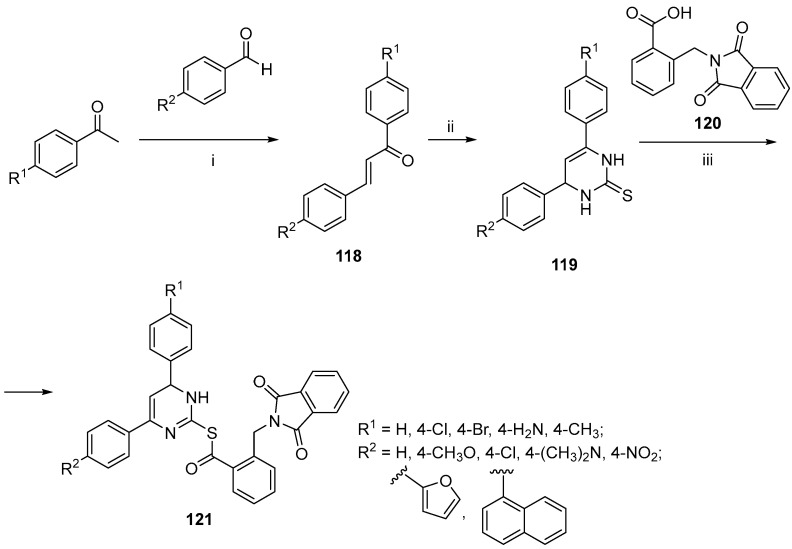
Reagents and conditions: (i) NaOH, dil. EtOH, rt; (ii) NaOH, abs. EtOH, 66–76%; (iii) abs. dichloromethane, TEA, rt, 65–75%.

Mourad et al. developed novel dihydropyrimidine phthalimide hybrids as analogues of alogliptin and phthalimide-based selective DPP-IV inhibitors [108]. The synthetic protocol starts from appropriately substituted chalcones **118**, which reacted with thiourea to form 4,6-diaryl-3,4-dihydropyrimidine-2(1H)-thiones **119**. The reaction with α-phthalimido-o-toluyl chloride **120** afforded the target compounds **121** (Scheme 40).

#### 3.2.5. 1,4-Dioxane

**Scheme 41:**
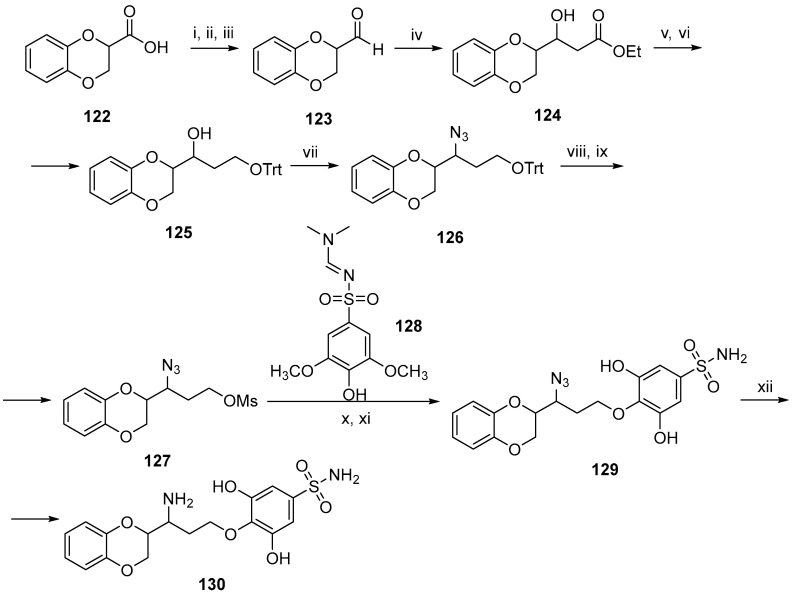
Reagents and conditions: (i) SOCl_2_, DCM, reflux; (ii) *N,O*-dimethylhydroxylamine, DCM, rt, 47% over two steps; (iii) LiAlH_4_, THF, −20 °C, quantitative yield; (iv) Zn, TBDMSiCl, ethyl bromoacetate, THF, reflux, 89%; (v) LiAlH_4_, THF, −10 °C, 48%; (vi) TrtCl, TEA, DCM, rt, 96%; (vii) PPh_3_, diethyl azodicarboxylate (DEAD), diphenylphosphorylazide (DPPA), THF, rt, 32%; (viii) Amberlist 15, DCM/MeOH, reflux, 41%; (ix) MsCl, TEA, DCM, rt, 92%; (x) K_2_CO_3_, *N,N*-dimethylformamide (DMF), 90 °C, 74%; (xi) NH_2_NH_2_·H_2_O, MeOH; (xii) NH_2_NH_2_·H_2_O, PdO, MeOH, reflux, 83%.

Artasensi et al. made a significant advancement in overcoming the limitations of existing multidrug therapies by developing ligands that inhibit both DPP-IV and carbonic anhydrases [109]. The synthetic methodology for their most potent compounds starts with 1,4-benzodioxan-2-carboxylic acid (**122**), which is converted to the corresponding aldehyde **123** via a three-step sequence (Scheme 41). The β-hydroxyester derivative **124** was obtained via the Reformatsky reaction of **123** with ethyl bromoacetate. Subsequent reduction of the ester group to give primary alcohol, followed by its protection with trityl chloride, afforded **125**. The Mitsunobu reaction of **125** gave the azide derivative **126**, which, upon deprotection and mesylation, yielded the key intermediate **127**. Nucleophilic substitution reaction with **128**, followed by sulfonamide deptorection, afforded **129**, which, after azide reduction, gave the target compound **130**.

### 3.3. Six-Membered Heterocycles with Three Heteroatoms and Their Benzo-Fused Derivatives

#### Triazine

**Scheme 42:**
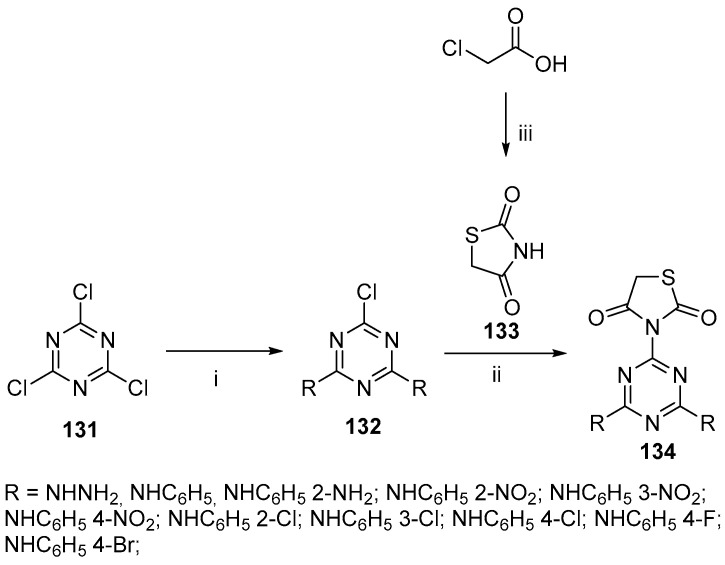
Reagents and conditions: (i) RNH_2_, NaOH, 40–45 °C, 64–87%; (ii) K_2_CO_3_, reflux, 120–135 °C; (iii) conc. H_2_SO_4_, H_2_O, reflux, 80%.

1,3,5-Triazines coupled with thiazolidine-2,4-diones have been shown to possess both antibacterial properties and DPP-IV-inhibitory activity [110]. The compounds were prepared using 2,4,6-trichloro-1,3,5-triazine (**131**), which reacted with amines to form disubstituted amino-1,3,5-triazine **132**. The latter reacted with thiazolidine-2,4-dione **133**, yielding the final products **134** (Scheme 42).

A similar synthetic methodology, starting with 2,4,6-trichloro-1,3,5-triazine **131**, has been used to yield 1,3,5-triazine derivatives containing sulfonamide [111] and sulfonamide and morpholine functionalities [112]. Recently, Gupta et al. obtained morpholine-1,3,5-triazine derivatives containing an additional five- or six-membered heterocycle (pyrimidine, pyrazole) [113,114,115].

## 4. Polycyclic Fused Heterocycles

### 4.1. Bicyclic 5-5 Systems

**Figure 5:**
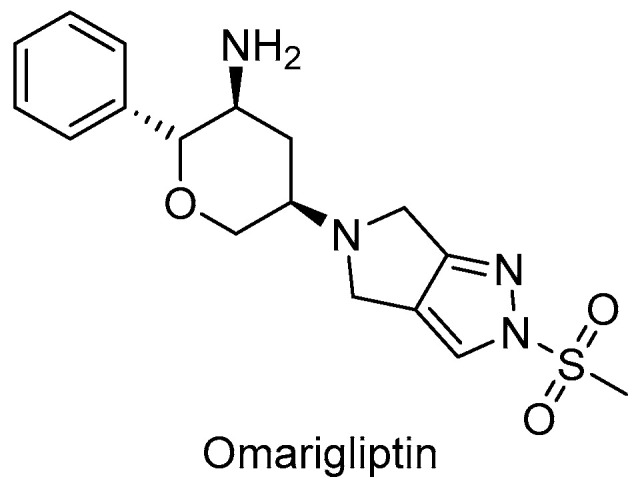
Structure of Omarigliptin.

A prominent example of fused five-membered heterocycles in the structure of clinically approved DPP-IV inhibitor is the drug Omarigliptin (Figure 5) [116,117].

**Scheme 43:**
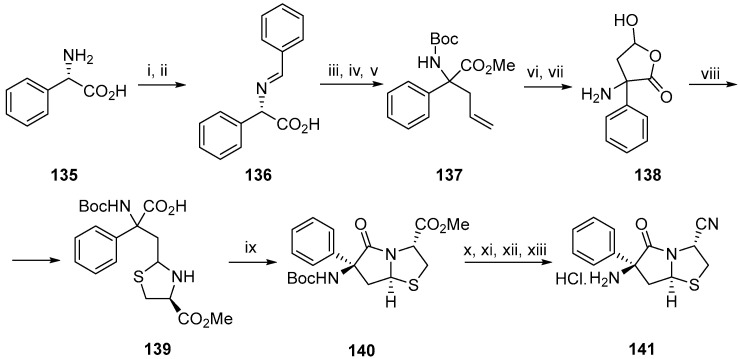
Reagents and conditions: (i) SOCl_2_, MeOH, 0 °C, 2 h, 89%; (ii) PhCHO, TEA, MgSO_4_, CH_2_Cl_2_, rt, 18 h, 97%; (iii) *t*-BuOK, CH_2_CHCH_2_Br, THF, rt, 18 h; (iv) 6 N HCl, EtOAc, 1 h; (v) Boc_2_O, THF, reflux, 18 h, 87%; (vi) O_3_, PPh_3_, CH_2_Cl_2_, −78 °C, 36%; (vii) LiOH, THF, 1 h, 95%; (viii) *L*-Cys-OMe·HCl, NaHCO_3_, EtOH, 16 h, 51%; (ix) *p*-TsOH, toluene, reflux, 1 h, 23%; (x) NH_3_, MeOH, rt, 2 h, 97%; (xi) TFAA, TEA, CH_2_Cl_2_, rt, 1 h, 84%; (xii) TFA, CH_2_Cl_2_, rt, 1 h, 14% after preparing HPLC; (xiii) HCl (g), CH_2_Cl_2_/Et_2_O, 2 h, 53%.

Betancort et al. developed a series of pyrrolo[2,1-b]thiazole derivatives starting with (*S*)-2-amino-2-phenylacetic acid **135**, which was methylated and converted to benzaldimine **136** (Scheme 43) [118]. After alkylating **136** with allyl bromide, and replacing the benzaldimine fragment with a Boc protecting group, the intermediate **137** was obtained. The C=C bond in **137** underwent ozonolysis and reductive work-up, resulting in the cyclic **138**. The thiazolidine ring was formed after the condensation of **138** with *L*-cysteine methyl ester as a mixture of diastereomers **139**, and after refluxing in toluene with *p*-toluene sulfonic acid, the additional pyrrolidine ring in **140** was formed. After deprotecting the amine group and converting the ester to nitrile, the target **141** was formed.

**Scheme 44:**
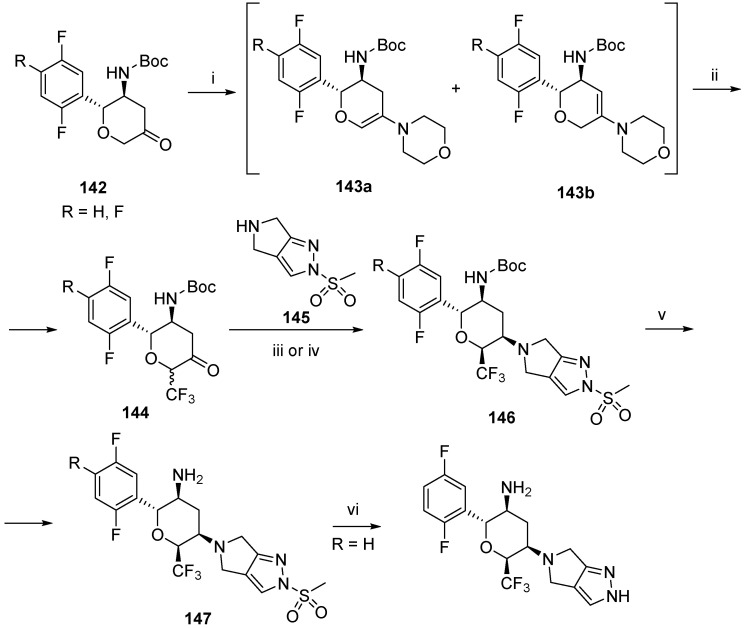
Reagents and conditions: (i) morpholine, toluene, reflux, Dean–Stark apparatus; (ii) Umemoto’s reagent (*S*-(trifluoromethyl)dibenzothiophenium trifluoromethanesulfonate), DMAP, DMAc, 57% over two steps; (iii) toluene, reflux, then NaBH(OAc)_3_, CH_3_COOH, 1,2-dichloroethane, 72%; (iv) CHCl_3_, reflux, Dean-Stark apparatus, then NaBH(OAc)_3_, CH_3_COOH, 1,2-dichloroethane, 72–75%; (v) TFA, DCM, 77–90%; (vi) *t*-BuOK, THF, 60%.

Zhang et al. developed analogies of Omarigliptin bearing fluorine or CF_3_-substituted tetrahydropyran rings (Scheme 44) [119]. The synthetic strategy begins with the tetrahydropyran **142**, which reacts with morpholine to produce a mixture of enamine derivatives **143a** and **143b**, which then reacts with Umemoto’s reagent to obtain the trifuoromethylated ketone **144**. After reductive amination of **144** with 2-(methylsulfonyl)-2,4,5,6-tetrahydropyrrolo[3,4-c]pyrazole (**145**), the target **146** was formed. The final Boc deprotection resulted in the lead compound **147**. Additionally, *t*-BuOK treatment of **147** leads to cleavage of the methylsulfonyl group.

### 4.2. Bicyclic 5-6 Systems

**Figure 6:**
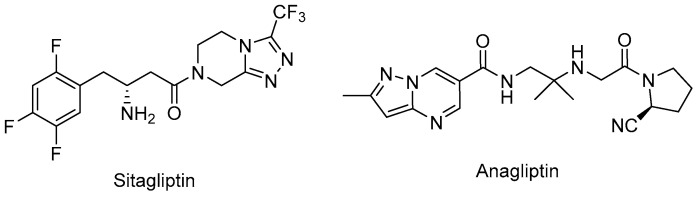
Structure of Sitagliptin and alogliptin.

Two prominent examples of DPP-IV inhibitors featuring bicyclic 5-6 heterocycles are Sitagliptin and Anagliptin, respectively (Figure 6).

**Scheme 45:**
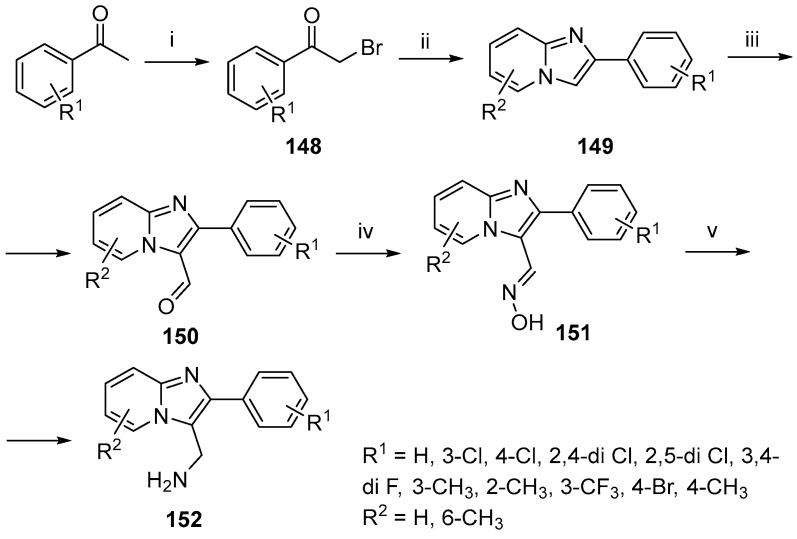
Reagents and conditions: (i) CuBr_2_, ethyl acetate, CHCl_3_, reflux, 16 h, 90%; (ii) 2-aminopyridine, NaHCO_3_, EtOH, rt, 80–84%; (iii) POCl_3_, DMF, 0–60 °C, 2 h, 65–68%; (iv) NH_2_OH. HCl, pyridine, EtOH, reflux, 83–90%; (v) Zn, NaOAc, AcOH, rt, 10 h, then Boc_2_O, NaOH, EtOH, rt, 3 h, then HCl(gas), ethyl acetate/ether, 0 °C, 36–71%.

One of the simplest bicyclic 5-6 heterocyclic systems that can be found in DPP-IV inhibitors is the imidazo[1,2-a]pyridine. Qing Lie et al. synthesized imidazo[1,2-a]pyridines and evaluated their DPP-IV-inhibitory activity [120]. The key ring-forming step is a reaction between 2-aminopyridine and phenacetyl bromides **148** (Scheme 45). The resultant **149** is treated with the Vismeier reagent to produce the aldehyde **150**, followed by condensation with hydroxylamine hydrochloride to form the oxime **151**. After reducing **151** with zinc powder in acetic acid, the resultant amine group was Boc-protected for the purposes of purification, and after final deprotection, the target **152** was obtained.

**Scheme 46:**
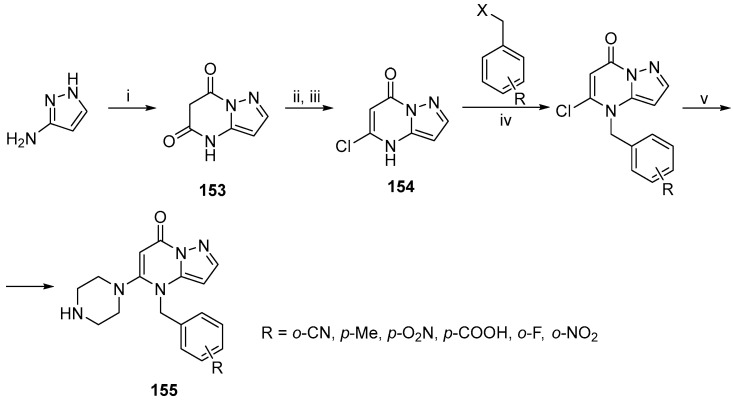
Reagents and conditions: (i) diethyl malonate, EtONa EtOH, reflux, 71%; (ii) POCl_3_, *N,N*-dimethyl-aniline, 100 °C, 80%; (iii) 1 N NaOH, 90 °C; (iv) Cs_2_CO_3_ or K_2_CO_3_ or DIPEA, DMF, 90 °C, 46–69%; (v) DIPEA, DMF, 90 °C, 20–33%.

Another heterocyclic system found in compounds that have a variety of interesting biological properties is the pyrazolo[1,5-a]pyrimidine ring system [121,122,123]. Deng et al. synthesized novel pyrazolo[1,5-a]pyrimidin-7(4*H*)-one derivatives as DPP-4 inhibitors that exhibit high potency and selectivity (Scheme 46) [124]. The synthetic methodology begins with 1*H*-pyrazol-5-amine, which reacts with diethyl malonate to produce the intermediate **153**. Chlorination with POCl_3_ and selective alkaline hydrolysis furnished the necessary chloro derivative **154**. Alkylation of **154** with benzyl halide derivatives and substitution of the chlorine atom gave the prospective inhibitors **155**.

Further research by the authors resulted in the development of DPP-IV inhibitors with increased potency compared to **155** by replacing the piperazine fragment in position 5 with (*R*)-piperidin-3-amine. The synthesis follows the methodology specified in Scheme 46 [125].

**Scheme 47:**
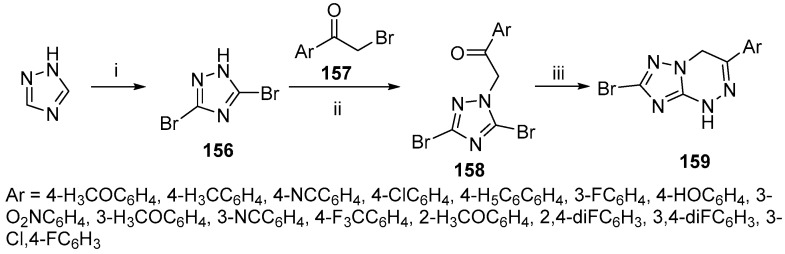
Reagents and conditions: (i) H_2_O, KHCO_3_, Br_2_ in aqueous solution of KBr, stirring, 30–40 min, 80 °C, 71%; (ii) K_2_CO_3_, stirring, 4–6 h, rt, 68–85%; (iii) NH_2_NH_2_·H_2_O 80%, MeOH, reflux, 8–9 h, 62–76%.

Patel et al. developed a series of triazolotriazine derivatives as novel DPP-IV inhibitors based on in silico studies and utilizing Sitagliptin as a reference compound [126]. After bromination of 1,2,4-triazole, the dibromo derivative **156** was obtained, and after *N*-alkylation with different phenacetyl bromides **157**, the substituted derivative **158** was formed. A final ring-closing reaction in which **158** reacted with hydrazine hydrate and subsequently formed the target **159** after nucleophilic substitution (Scheme 47).

**Scheme 48:**
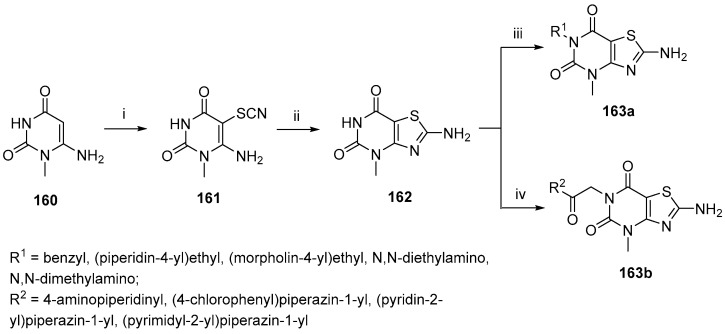
Reagents and conditions: (i) Br_2_/KSCN, DMF, 83%; (ii) DMF, 80–120 °C, 89%; (iii) R^1^Cl, anhydrous K_2_CO_3_, DMF, 70 °C, 74–78% (iv) *N*-substituted chloracetamides, anhydrous K_2_CO_3_, DMF, 70–80 °C, 64–68%.

Another heterocyclic fragment that can be found in DPP-IV inhibitors is the thiazolopyrimidine ring system [127]. Sharma et al. reported the synthesis, in silico, and in vitro evaluation of thiazolopyrimidine derivatives as DPP-IV inhibitors, as well as their structure–activity relationship (Scheme 48) [128]. The core heterocyclic structure was formed in two steps, beginning with the 6-amino pyrimidine derivative **160**. First, a thiocyanate group was added on C-5, and a cyclization reaction of **161** to give 2-amino-4-methyl-4*H*-thiazolo [4,5-d]pyrimidine-5,7-dione **162**. The target compounds **163a**–**b** were prepared via *N*-alkylation of **162** with alkyl halides of *N*-substituted chloracetamides.

### 4.3. Bicyclic 6-6 Systems

**Scheme 49:**
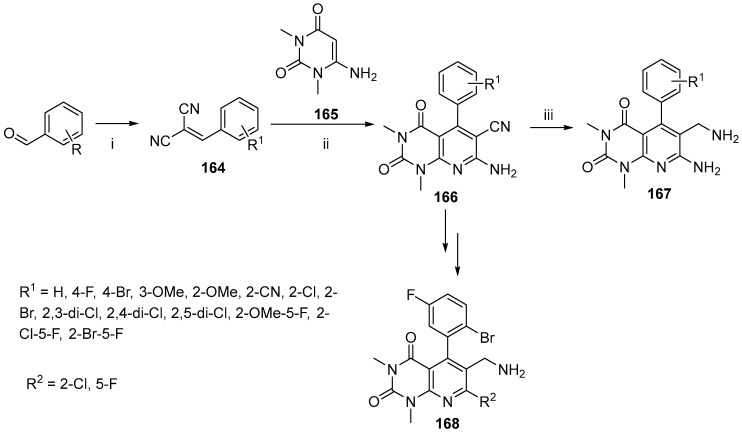
Reagents and conditions: (i) malonodinitrile, ethanol, 10% aq KOH, 95%; (ii) propanol, 120 °C, 43%; (iii) BH_3_-THF, THF, 65 °C, 46%.

An important example of a bicyclic system of two fused six-membered heterocycles that has found application in DPP-IV inhibitors is the pyridopyrimidine ring system. It was first described by Betty Lam et al. as a potent and selective DPP-IV inhibitor in 2012 [129]. The synthesis of the compounds started with a Knoevenagel condensation of substituted aromatic aldehydes with malonodinitrile to generate the derivatives **164**, which, upon reaction with aminopyrimidine **165**, led to the formation of the core heterocycle **166** via a Michael addition reaction (Scheme 49). BH_3_-mediated reduction of the nitrile group resulted in the formation of **167**, while compounds **168** resulted from a three-step synthetic procedure.

**Scheme 50:**
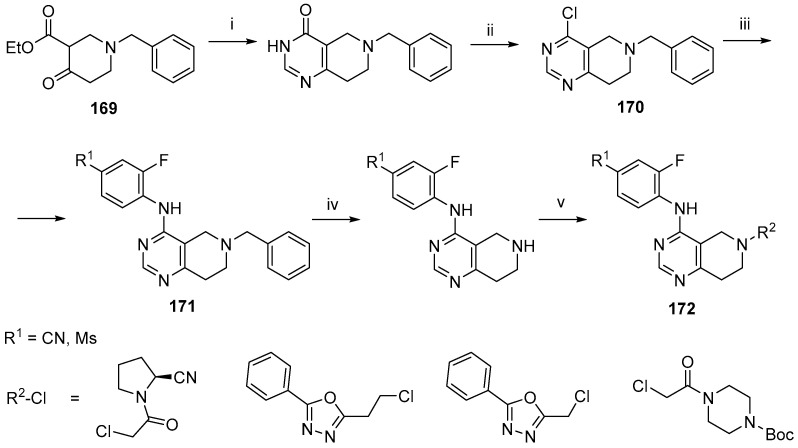
Reagents and conditions: (i) formamide, NaOEt, EtOH, reflux; (ii) POCl_3_, reflux; (iii) 4-amino-3-fluorobenzonitrile, NaH, THF, reflux, overnight, 35% or 2-fluoro-4-(methylsulfonyl)aniline, X-Phos, Pd_2_(dba)_3_, Cs_2_CO_3_, dioxane, reflux, overnight, 83%; (iv) 2-chloroethyl chloroformate, 1,2-dichloroethane, reflux, overnight, then MeOH, reflux, 3 h, 76%; (v) R^2^Cl, K_2_CO_3_, DMF, 70 °C, overnight, 64–94%.

Fang et al. conducted research on novel tetrahydropyridopyrimidine derivatives that exhibit both GPR119 and DPP-IV-inhibitory activity [130]. The authors synthesized two series of compounds—based either on 4,6-disubstituted tetrahydropyrido[4,3-d]pyrimidine or on 4,7-disubstituted tetrahydropyrido[3,4-d]pyrimidine scaffold. The synthetic methodology for the 4,6-disubstituted tetrahydropyrido[4,3-d]pyrimidine derivatives is outlined in Scheme 50, as the synthesis of both series follows similar steps. The procedure starts with commercially available 1-benzyl-3-carbethoxy-4-piperidone **169**, which cyclizes with formamide, followed by chlorination with POCl_3_ to give **170** [131]. The substitution of chlorine with 2-fluoro-4-cyanoaniline or 2-fluoro-4-methylsulfonyl aniline gives **171**. Deprotection of the benzyl group in **171**, followed by *N*-alkylation, afforded the final **172**.

### 4.4. Tricyclic Systems

**Scheme 51:**
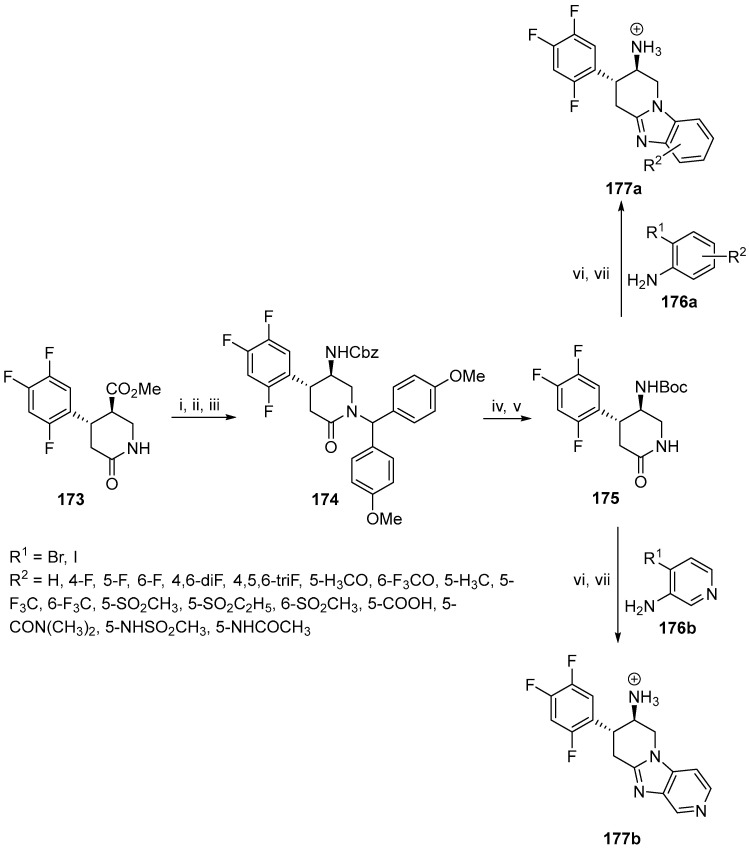
Reagents and conditions: (i) 4,4′-dimethoxybenzhydrol, H_2_SO_4_, HOAc, 47%; (ii) LiOH(aq), THF, 80%; (iii) DPPA, TEA, toluene, BnOH, reflux, 65%; (iv) Pd(OH)_2_, H_2_, MeOH, Boc_2_O, 73%; (v) ceric ammonium nitrate, CH_3_CN, H_2_O, 0 °C, 75%; (vi) CuI, MeNHCH_2_CH_2_NHMe, K_2_CO_3_, toluene, reflux; (vii) TFA, CH_2_Cl_2_, 33–80% over two steps.

An important example of the use of a tricyclic system in DPP-IV inhibitors is the research by Emondson et al. on piperidine-fused benzimidazoles and imidazopyridines, which are described as potent inhibitors in nanomolar concentrations [132]. The synthetic route starts with the cyclic lactam **173**, which is protected with 4,4′-dimethoxybenzhydrol and then converted to **174** via ester hydrolysis and Curtius rearrangement (Scheme 51). Exchange of Cbz with the Boc-protecting group and deprotection of lactam nitrogen gave **175**, which was used in the key ring-forming reaction with various aromatic halogen-substituted amines **176a**–**b** to furnish the target inhibitors **177a**–**b**.

**Scheme 52:**
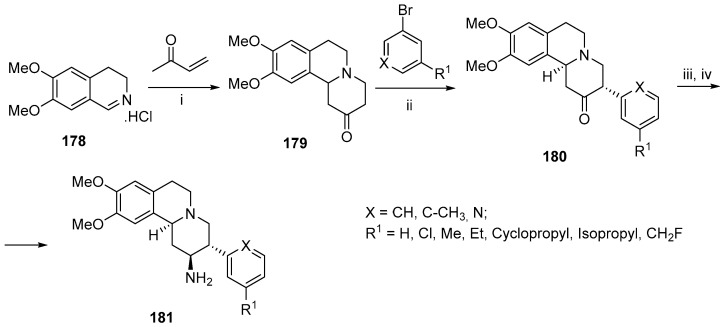
Reagents and conditions: (i) water bath, 1 h, then 5% Na_2_CO_3_; (ii) Pd(OAc)_2_, PtBu_3_, *t*-BuONa, THF, 21–51%; (iii) NH_2_OH.HCl, NaOAc, EtOH, 83–99%; (iv) H_2_, Raney Ni, aq NH_3_, MeOH, THF, 34–85%.

Boehringer et al. demonstrated the promising DPP-IV-inhibitory activity of benzo[a]quinolizidine derivatives with IC_50_ values in the nanomolar range [133]. The authors employed a four-step synthetic route starting with the 3,4-dihydroisoquinoline derivative **178**, which reacted with methyl vinyl ketone to form the key intermediate **179** based on a previously reported procedure [134]. The ketone **179** underwent palladium-catalyzed arylation to give **180** as a single stereoisomer. The final step was the conversion of the carbonyl group to oxime, followed by reduction to give **181**, bearing an amino group at C-2 (Scheme 52). Further research by the authors led to the discovery of Carmegliptin, which is a highly potent DPP-IV inhibitor [135]. Other authors have also investigated benzo[a]quinolizidine compounds and their heterocyclic analogues as prospective DPP-IV inhibitors [136,137].

**Scheme 53:**
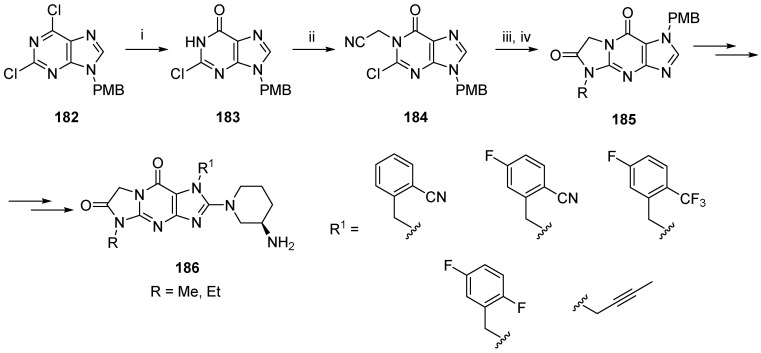
Reagents and conditions: (i) NaOH, 90%; (ii) BrCH_2_CN, *N,N*-Diisopropylethylamine, DMF, 55%; (iii) MeNH_2_ or EtNH_2_, 1,4-dioxane, rt; (iv) 6 N HCl, reflux, 65%.

The work of Wu et al. provides another example of potent tricyclic DPP-IV inhibitors [138]. The authors constructed novel tricyclic guanine derivatives starting with 2,6-dichloropurine **182** using selective hydrolysis to obtain **183**. Alkylation gave **184**, which was then reacted with methylamine or ethylamine, followed by refluxing with 6 N HCl to give the target heterocycle **185**. The obtained **185** was further used for the preparation of the prospective inhibitors **186** (Scheme 53).

## 5. Conclusions

This review is focused on heterocyclic scaffolds that have been the subject of research by several different research groups. As can be seen, there is a great structural diversity of heterocycles that could be found in DPP-IV inhibitors. However, the search for novel compounds with improved potency and selectivity is an ongoing challenge in contemporary medicinal chemistry. The development of DPP-IV inhibitors is a multidisciplinary field of research, supported by various in silico drug design approaches, innovative synthetic methodologies, and in vitro methods to assess bioactivity and toxicity.

## Data Availability

No new data were created or analyzed in this study. Data sharing is not applicable to this article.
